# VipariNama: RNA viral vectors to rapidly elucidate the relationship between gene expression and phenotype

**DOI:** 10.1093/plphys/kiab197

**Published:** 2021-05-02

**Authors:** Arjun Khakhar, Cecily Wang, Ryan Swanson, Sydney Stokke, Furva Rizvi, Surbhi Sarup, John Hobbs, Daniel F Voytas

**Affiliations:** 1 Department Genetics, Cell Biology, & Development, University of Minnesota, Minneapolis 55108, USA; 2 Center for Precision Plant Genomics, University of Minnesota, St Paul, Minneapolis 55108, USA

## Abstract

Synthetic transcription factors have great promise as tools to help elucidate relationships between gene expression and phenotype by allowing tunable alterations of gene expression without genomic alterations of the loci being studied. However, the years-long timescales, high cost, and technical skill associated with plant transformation have limited their use. In this work, we developed a technology called VipariNama (ViN) in which vectors based on the tobacco rattle virus are used to rapidly deploy Cas9-based synthetic transcription factors and reprogram gene expression in planta. We demonstrate that ViN vectors can implement activation or repression of multiple genes systemically and persistently over several weeks in *Nicotiana benthamiana*, Arabidopsis (*Arabidopsis thaliana*), and tomato (*Solanum lycopersicum*). By exploring strategies including RNA scaffolding, viral vector ensembles, and viral engineering, we describe how the flexibility and efficacy of regulation can be improved. We also show how this transcriptional reprogramming can create predictable changes to metabolic phenotypes, such as gibberellin biosynthesis in *N. benthamiana* and anthocyanin accumulation in Arabidopsis, as well as developmental phenotypes, such as plant size in *N. benthamiana*, Arabidopsis, and tomato. These results demonstrate how ViN vector-based reprogramming of different aspects of gibberellin signaling can be used to engineer plant size in a range of plant species in a matter of weeks. In summary, ViN accelerates the timeline for generating phenotypes from over a year to just a few weeks, providing an attractive alternative to transgenesis for synthetic transcription factor-enabled hypothesis testing and crop engineering.

## Teaser

RNA vectors systemically deliver synthetic transcription factors to a range of plants to rapidly elucidate the relationship between gene expression and phenotype without transgenesis.

## Introduction

There is an urgent need for tools that accelerate the pace of biological discovery in crop plants, so mechanistic insights can be leveraged to enhance crops and help deliver global food security (Fanzo et al., 2018; Soares et al., 2019). By examining the genomic changes underlying the domestication of wild plants into modern crops, it has become clear that many desirable traits are driven by changes in the expression of certain key genes (Cubillos et al., 2012; Eshed and Lippman, 2019). These insights highlight how mechanistic models that relate gene expression to development and metabolism could be used to determine the changes in gene expression required to obtain agriculturally beneficial phenotypes.

Building and validating robust mechanistic models of how gene expression relates to the whole plant phenotype relies on the ability to study the phenotypic outcome of controlled changes to gene expression. These changes could be implemented in a single step using synthetic transcription factors with programmable DNA-binding domains, such as Cas9 (Khakhar et al., 2018). These tools allow the exploration of intermediate ranges of gene expression between classical overexpression and knockout. They also have certain benefits over other possible interventions, for example, as they are able to layer additional regulation onto the native locus. This is in contrast to strategies that complement knockout lines with copies of the gene being studied driven by constitutive or inducible promoters inserted in novel genomic contexts. Synthetic transcription factors also enable both tunable activation and repression of genes, unlike approaches such as viral-induced gene silencing (VIGs) or RNA interference, which can only implement repression without an obvious way to tune the strength of regulation. However, the benefits of synthetic transcription factors come with the drawback of having to compete with endogenous transcription factors, which can sometimes lead to modest changes in expression. Synthetic transcription factors have previously been used to make predictable alterations in expression in a range of plants (Piatek et al., 2014; Khakhar et al., 2018; Selma et al., 2019). Their use for validation of mechanistic models of plant development was first demonstrated in Arabidopsis (*Arabidopsis thaliana*), where a mathematical model of auxin-regulated branching was validated through the deployment of a hormone-activated Cas9-based repressor (HACR) to reprogram shoot architecture (Khakhar et al., 2018).

**Figure 1:**
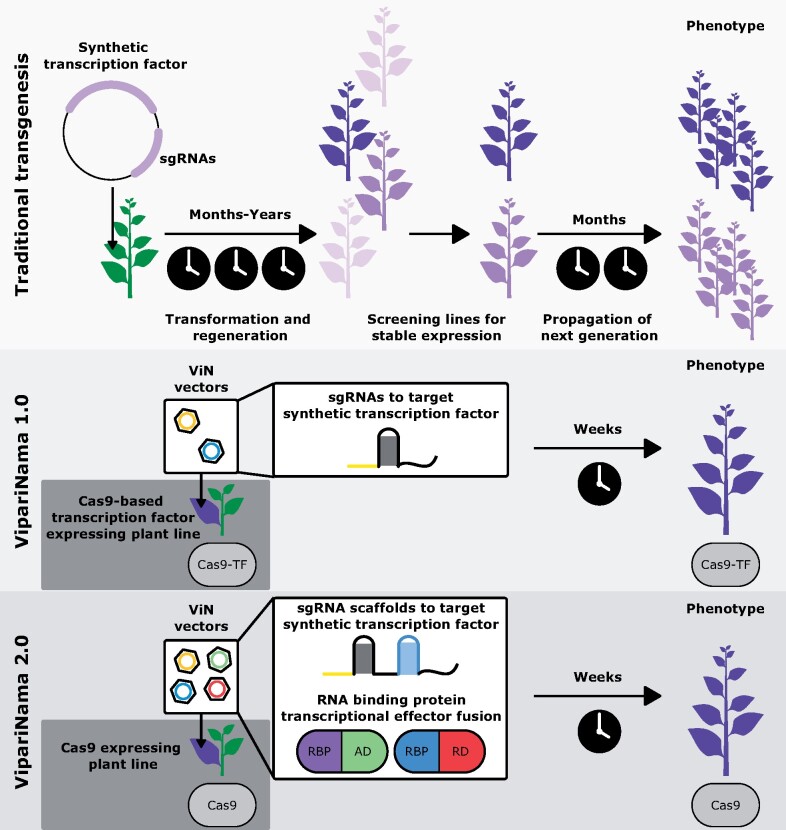
ViN allows rapid reprograming of plant morphology and metabolism by delivering synthetic transcription factors on RNA vectors. Schematics depicting how synthetic transcription factors can be deployed to perturb gene expression and create phenotypes via traditional transgenesis (top), ViN 1.0 (middle), where ViN vectors deliver sgRNAs to a plant that is stably expressing Cas9-based transcription factors, and ViN 2.0 (bottom), where ViN vector ensembles deliver both sgRNA scaffolds as well as RNA-binding protein-transcriptional effector fusions. TF, transcription factor; RBP, RNA-binding protein; AD, activation domain; RD, repression domain.

While this work provided a proof-of-concept for deployment of synthetic transcription factors for the elucidation of biological mechanisms, the extension of this strategy into crop plants faces a hurdle shared by most applications of plant synthetic biology: the challenges of generating transgenic plants. These challenges include high costs and technical skill, which restrict access to high-resource settings, as well as a sometimes year-long time scale, slowing the iterative design–build–test cycle of synthetic biology to a crawl (Altpeter et al., 2016). In this work, we sought to circumvent this core challenge with a new platform called VipariNama (ViN), the Sanskrit word meaning “to change” (Figure 1). ViN uses RNA viral vectors (ViN vectors), which can spread throughout the plant and persist over time, to reprogram synthetic transcription factors and make alterations to gene expression, and thereby rapidly engineer phenotypes without necessitating transgenesis after the initial establishment of a line expressing the transcription factor components. This approach conceptually parallels the use of RNA vectors in gene therapies (Walther and Stein, 2000), but with the end goal of re-engineering plant metabolism and morphology to elucidate or validate mechanistic models of biology.

Positive single-stranded RNA viruses are an ideal starting point to build ViN vectors from, as they are systemically mobile and persist for long periods of time in plants (Cody and Scholthof, 2019). Some of these viruses can have very broad host ranges and are largely asymptomatic. For these reasons, we decided to base our ViN vectors on the Tobravirus, tobacco rattle virus (TRV; Cody and Scholthof, 2019). Previous work has identified regions of the TRV genome into which foreign gene sequences can be added to re-purpose these viruses as protein production tools (Larsen and Curtis, 2012). However, using the same strategy to deliver synthetic transcription factors, such as the HACR, is not possible due to the limited loading capacity of the virus, namely <1 kb (Pasin et al., 2019). Larger cargos abrogate the viruses’ capacity for systemic movement through the plant and are often lost through recombination.

To overcome this challenge, we developed ViN 1.0, wherein we created a transgenic plant constitutively expressing a Cas9-based synthetic transcription factor and used ViN vectors to deliver single guide RNAs (sgRNAs) to specify targets for this transcription factor (Figure 1). Next, we demonstrated how this strategy can be made more flexible using sgRNA scaffolds to simultaneously generate activation and repression-based phenotypes in a plant. Finally, we showed how this platform could be made even more flexible by building an ensemble of ViN vectors to deliver nearly all the synthetic transcription factor components to a plant stably expressing Cas9. This system, ViN 2.0, can implement persistent activation or repression of multiple genes across several plant species. We also demonstrated ViN vectors can be used to rapidly create metabolic and morphological phenotypes. As a proof of concept, we explored if qualitative predictions from models of gibberellin (GA) signaling (Middleton et al., 2012) could be rapidly validated using ViN vectors to perturb gibberellin signaling and associated plant size, an agriculturally important trait, in the model plants Arabidopsis and *Nicotiana benthamiana*, as well as the crop, tomato (*Solanum lycopersicum*).

## Results

### ViN vectors deliver sgRNAs to GA-HACR *N. benthamiana* lines and implement GA-responsive repression

**Figure 2:**
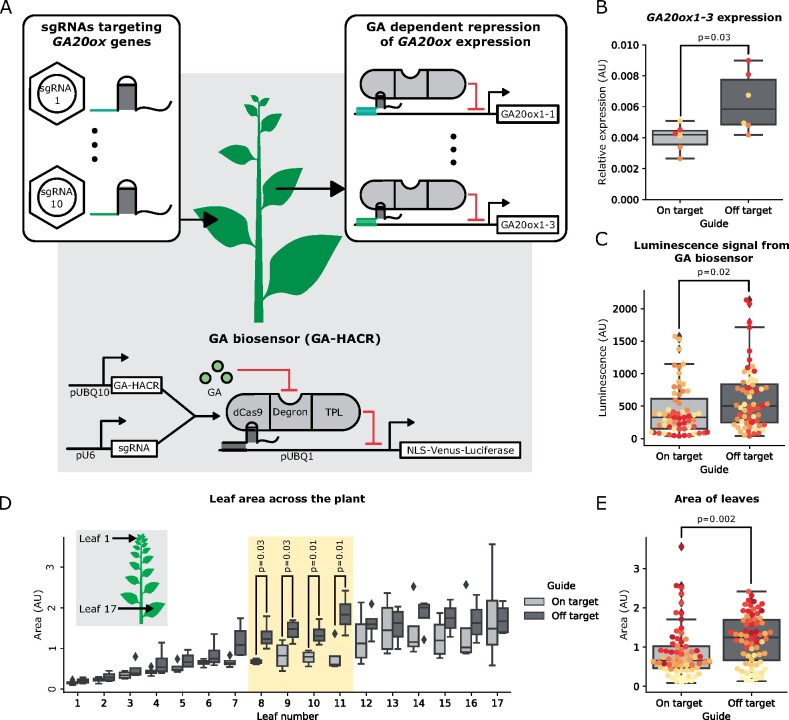
ViN vectors can deliver sgRNAs to reprogram a HACR to repress *GA20ox* expression and create a reduction in GA levels and an associated reduction in leaf area. A, Schematic describing the GA-HACR-based GA biosensor that is integrated into the *N. benthamiana* genome. Here, the constitutively expressed GA-HACR complexes with a sgRNA that targets it to repress a luciferase reporter in a GA-dependent manner. This reporter creates a luminescence signal in response to GA in a dose-dependent manner. The inset panels show ViN vectors delivering sgRNAs to target the GA-HACR to the *GA20ox* genes in *N. benthamiana* (left) and resultant reprogrammed HACRs in systemic tissues (right). B, Box plots summarizing relative expression of *GA20ox1-3*, normalized to the *EF-1 alpha* housekeeping gene, from systemic leaves of the plant lines described in (A) that were treated with ViN vectors encoding on-target (light gray) or off-target (dark gray) sgRNAs. In the boxplots for all panels in this figure, the horizontal line through the box marks the median, the box is the range from the 25th to the 75th percentile, and the whiskers show the highest and lowest data points within 1.5 times the interquartile range. Each dot of the same color represents data from independent biological replicates (*n* = 3 per treatment). Reported *P*-values were calculated using a *t* test; *P* < 0.05 was considered significant. C, Box plots summarizing luminescence signal of the GA biosensor from the leaves of plants treated with ViN vectors encoding on-target (light gray) or off-target (dark gray) sgRNAs. Each dot of the same color represents data from leaves on an independent biological replicate (*n* = 4 per treatment). Reported *P*-values were calculated using a *t* test; *P* < 0.05 was considered significant. D, Box plots summarizing the area of leaves of the plants described in (A) treated with ViN vectors encoding on-target (light gray) or off-target (dark gray) sgRNAs. The inset shows all the leaves assayed across the plant, with leaf number 1 being the top leaf. The yellow region highlights the leaves in which a statistically significant difference in size between plants treated with on-target and off-target sgRNAs was observed. E, Box plots summarizing the area of all the leaves plotted together. Each dot represents data from a leaf of an independent biological replicate (*n* = 4 per treatment). Reported *P*-values were calculated using a *t* test; *P* < 0.05 was considered significant. NLS, nuclear localization signal; TPL, TOPLESS.

Previous characterization of the GA-HACR, a GA-responsive Cas9-based repressor, demonstrated it is an effective tool to study GA signaling in planta (Khakhar et al., 2018). HACRs consist of a deactivated Cas9 protein (dCas9) fused to a phytohormone degron and a transcriptional repression domain. When complexed with a co-expressed sgRNA, a HACR is targeted to repress expression of a specific gene. In the presence of a threshold phytohormone concentration, the HACR is targeted for degradation, thus relieving repression. In lines that have a stably integrated GA-HACR biosensor, a significant increase in luciferase signal is observed in response to external GA treatment or internal GA biosynthesis (Khakhar et al., 2018). As the TRV parent virus for ViN vectors has been previously shown to propagate well in *N. benthamiana* (Velásquez et al., 2009), we created transgenic lines of this species constitutively expressing a GA-HACR and used these plants to test the capacity of ViN vectors to reprogram transcription (Figure 2A). Our aim was to target the GA-HACR to repress the expression of the *Gibberellin 20-oxidase* (*GA20ox*) genes in the transgenic lines. These genes are responsible for GA biosynthesis, and the negative feedback loop created by their GA-dependent reduction in expression is an important parameter controlling the concentration of GA in the cell (Middleton et al., 2012).

ViN vectors were built to encode sgRNAs targeting two sites within the first 500-bp upstream of the transcription start sites of five putative *GA20ox* genes. These vectors were delivered via *Agrobacterium* (*Agrobacterium tumefaciens*) infiltration to young plants in parallel with control vectors that encoded sgRNAs with no specific targets in the *N. benthamiana* genome. We used this same strategy for on- and off-target sgRNA design, as well as vector delivery, in all the other experiments described in this work.

RNA was extracted from tissue from the fourth leaf above the infiltrated leaf (the fourth systemic leaf), 24 d after vector delivery and expression of the target genes was quantified with quantitative reverse transcription polymerase chain reaction (RT-qPCR). We observed significant repression of the expression of the *GA20ox1-3* gene in plants treated with ViN vectors encoding the on-target sgRNAs compared to plants treated with the off-target control (Figure 2B). We also observed a decrease in the median expression of the two more highly expressed *GA20ox* genes targeted, *GA20ox1-1* and *GA20ox1-2*; however, the effect was not significant (Supplemental Figure S1). This might be because these strongly expressed genes are more challenging to repress (Selma et al., 2019). The other two GA20ox genes we targeted, *GA20ox1D-1* and *GA20ox1D-2*, were not expressed in the leaves at significant levels and so we would not expect to observe repression (Supplemental Figure S1). These results demonstrate how ViN vectors can be applied to reprogram synthetic Cas9-based transcription factors by delivering the appropriate sgRNAs. These results also show that the transcriptional regulation conferred by this approach can be observed in systemic leaves for several weeks post-delivery.

### ViN vector-mediated transcriptional reprogramming in GA-HACR plant lines generates predicted metabolic and developmental phenotypes

The GA-HACR line we built incorporates the previously described GA-biosensor (Figure 2A). As the GA-HACR is targeted to regulate a luciferase reporter, also included in this construct, in a GA-dependent manner, treatment of these plants with luciferin substrate should result in a luciferase signal proportional to the concentration of GA within that tissue (Khakhar et al., 2018). We used this reporter to compare GA levels in GA-HACR *N. benthamiana* plants to which we had delivered sgRNAs targeting the *GA20ox* gene family, the GA biosynthesis genes, and off-target controls.

The expression of *GA20ox* has been described to be regulated by GA concentration in the cell through a negative feedback loop, with higher GA concentrations leading to the degradation of the DELLA proteins, and thereby reduced activation of *GA20ox* expression and in turn reduced GA biosynthesis. By adding a layer of GA-dependent repression on *GA20ox* expression via the GA-HACR, essentially the opposite of the GA-dependent activation of the DELLA, we would expect to reduce the strength of the negative feedback between *GA20ox* expression and GA concentrations. Based on a model of the GA signaling pathway, the reduction in negative feedback and shift towards a more constitutive rather than dose-dependent *GA20ox* expression profile should result in a decrease in the cellular GA concentration and commensurate increase in DELLA protein concentrations (Middleton et al., 2012). Indeed, when we compared the dose–response relationship of *GA20ox* expression to exogenous GA application in plants treated with on target guides, we observe an ∼40% decrease in *GA20ox* expression at 3-h post-treatment with GA compared to an off target guide control where we observe an ∼60% decrease (Supplemental Figure S2). This is consistent with the predicted reduction in strength of negative feedback in *GA20ox* regulation. Using the integrated GA-HACR-based GA-biosensor, we observed a significant decrease in the luminescence observed from plants treated with on-target sgRNAs compared to the controls (Figure 2C). This is consistent with the prediction that a decrease in *GA20ox* expression should lower endogenous GA levels.

The reduction in GA levels brought on by a reduced negative feedback in *GA20ox* expression regulation should also result in an increased accumulation of DELLA proteins and an associated dwarfing in leaf tissue (Xiao et al., 2006; Band et al., 2012; Serrano-Mislata et al., 2017). We phenotyped plants treated with ViN vectors 5 weeks post infection and observed a significant decrease in leaf area in plants treated with on-target sgRNAs compared to plants treated with control sgRNAs (Figure 2E). This effect was particularly pronounced in the leaves closer to the infiltration site (leaf numbers 8–11; Figure 2D). We hypothesize this was because these leaves were infected early in development, unlike older leaves, and had sufficient time to fully expand by the time of measurement, in contrast to younger, still-developing leaves. These results were replicated in an independently grown set of plants, confirming that they were not due to growth conditions or relative plant health (Supplemental Figure S3).

### sgRNA scaffolds delivered via ViN vectors simultaneously create activation and repression phenotypes

**Figure 3:**
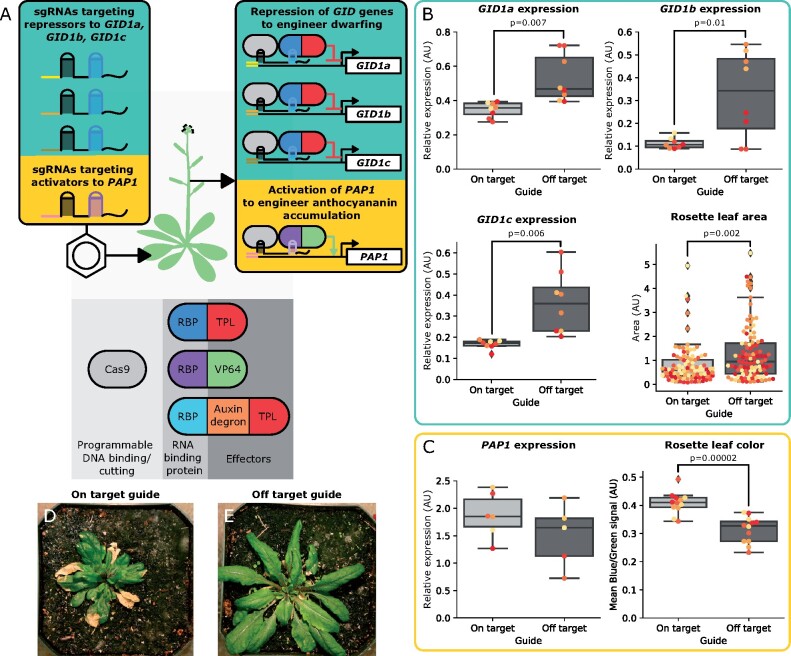
ViN vectors deliver sgRNA scaffolds to plant lines that enable scaffold-based reconstitution of transcription factors to implement transcriptional regulation and alter phenotypes. A, Schematic describing components expressed by an Arabidopsis line to enable scaffold-based reconstitution of transcription factors. This line constitutively expresses nuclease active Cas9 as well as three RNA-binding proteins fused to either a truncation of the TOPLESS repressor from Arabidopsis, the VP64 activator, or an auxin degron, which is also fused to a TOPLESS repressor. The colored insets describe the sgRNA scaffolds being delivered to this line via ViN vectors (left) and the resultant transcriptional perturbations as well as the expected phenotypic results (right). B, Box plots summarizing the expression of the *GID1a*, *GID1b*, and *GID1c* genes, normalized to the *PP2A* housekeeping gene, in rosette leaves as well as the rosette leaf area of the plants described in (A) that were treated with ViN vectors encoding on-target (light gray) or off-target (dark gray) sgRNAs. For all boxplots in this figure, the horizontal line through the box marks the median, the box is the range from the 25th to the 75th percentile, and the whiskers show the highest and lowest data points within 1.5 times the interquartile range. Each dot of the same color represents data from independent biological replicates (*n* = 4 per treatment). Reported *P*-values were calculated using a *t* test; *P* < 0.05 was considered significant. C, Box plots summarizing data collected from the rosette leaves of plants described in (A) that were treated with ViN vectors encoding on-target (light gray) or off-target (dark gray) sgRNAs 3 weeks after delivery. The box plot on the left summarizes the expression of the *PAP1* gene, normalized to the *PP2A* housekeeping gene, in rosette leaves. The box plot on the right summarizes the average blue signal normalized to the green signal, which is an established proxy for anthocyanin concentration (Yang et al., 2016), from images of rosette leaves. Each dot of the same color represents data from leaves of independent biological replicates (*n* = 5 per treatment for expression and *n* = 4 per treatment for leaf color). Reported *P*-values were calculated using a *t* test; *P* < 0.05 was considered significant. D, E, Representative pictures of rosettes of plants treated with on-target (D) or off-target (E) sgRNAs at the time of phenotyping. RBP, RNA-binding protein; TPL, TOPLESS; VP64, a transcriptional activator derived from Herpes Simplex Viral Protein 16.

One potential drawback of using HACR lines for reprogramming plant phenotypes is that certain traits require phytohormone independent regulation, or simultaneous activation and repression of multiple genes with different effectors. This would require an orthogonal dCas9 variant for each effector and the construction of a new transgenic line for each unique combination, thereby slowing down hypothesis testing. To overcome this limitation, we designed plant lines that enable scaffold-based reconstitution of transcription factors. Multiple constitutive expression cassettes are incorporated into these plant’s genome: a Cas9 cassette to provide programmable DNA binding and cutting, and a set of unique RNA-binding proteins fused to different transcriptional effectors (Figure 3A). ViN vectors can then be used to deliver specialized sgRNA scaffolds to these plants, which serve to direct Cas9 to a genomic target, but also to recruit specific transcriptional effectors. By truncating the target site of the sgRNA to 14 bp in these scaffolds, the nuclease-active Cas9 is directed to bind to DNA, but not cut it (Kiani et al., 2015). The addition of specific motifs at the 3′-end of the scaffold enables interaction with a specific RNA-binding protein fused to a transcriptional effector. Together, these components reconstitute the desired transcription factor at the locus of interest (Zalatan et al., 2015; Figure 3A). In principle, this would enable simultaneous activation and repression of different genes in planta. Delivering a full-length sgRNA, rather than a truncated sgRNA scaffold, to these same lines would allow targeted gene ablation, because the Cas9 nuclease is active. This strategy has been demonstrated previously for efficient somatic genome editing (Mahas et al., 2019).

To test this system, we built lines of Arabidopsis encoding the previously described components (Figure 3A). ViN vectors were then used to simultaneously deliver a set of sgRNA scaffolds targeting a repressor to the three *GIBBERELLIN-INSENSITIVE DWARF* (*GID*) genes, which are GA receptors (Middleton et al., 2012), and an activator to the *PRODUCTION OF ANTHOCYANIN PIGMENT 1* (*PAP1*) gene, which encodes a MYB transcription factor (Weigel et al., 2000; Lee et al., 2016; Figure 3A). Tissue was collected from systemic rosette leaves 24 d post-delivery. When we compared expression of the three *GID* genes in plants treated with on-target sgRNAs to controls we see a significant repression of all three genes (Figure 3B). Previous studies and the model of GA signaling both suggest that reducing *GID* expression should result in dwarfing, due to the increased concentration of growth-inhibiting DELLA proteins (Achard et al., 2009; Middleton et al., 2012). We observed a significant reduction of rosette leaf area in plants treated with on-target sgRNAs compared to plants treated with off-target controls (Figure 3, B, D and E).

We do not observe a statistically significant increase in the expression of the *PAP1* gene in plants treated with the on-target sgRNAs compared to controls (Figure 3C). This might be because the gene is natively highly expressed: *PAP1* expression in control plants is five times the *GID* expression levels, making any increase in expression subtle at best (Selma et al., 2019). However, as PAP1 is a master regulator of the anthocyanin biosynthesis pathway, even subtle changes in expression could result in a phenotype. Previous studies also show that the overexpression of *PAP1* results in anthocyanin accumulation (Weigel et al., 2000; Lee et al., 2016). The average ratio of blue to green signal in images of leaves is an established proxy for anthocyanin accumulation (Yang et al., 2016). We observed a significant increase in the average ratio of blue to green signal in the images of rosette leaves of plants treated with on-target sgRNAs compared to controls (Figure 3C). This phenotype was further confirmed through the extraction and quantification of anthocyanin from systemic leaves via spectrophotometry (Duarte et al., 2013). We observe significantly higher absorbance at 536 nm, the absorbance maximum of anthocyanin, in plants treated with on-target guides compared to controls (Supplemental Figure S4). These results imply that while the increase in *PAP1* expression is too subtle to see in the leaves assayed, it is still be capable of producing the increased accumulation of purple anthocyanin pigment. To demonstrate that these were two separate phenotypes, and not just two phenotypes caused by the observed *GID* repression, we repeated the experiment in a plant line without the repressor targeted to the *GID* genes. We again observed a significant increase in the average ratio of blue to green signal in images of rosette leaves, as well as an increased absorbance of extracts from these leaves at 536 nm, of plants treated with on-target sgRNAs, consistent with anthocyanin accumulation from increased *PAP1* expression (Supplemental Figures S4 and S5). This demonstrates that these are independent phenotypes.

### ViN vector ensembles can implement transcriptional regulation in Cas9-expressing plant lines

**Figure 4:**
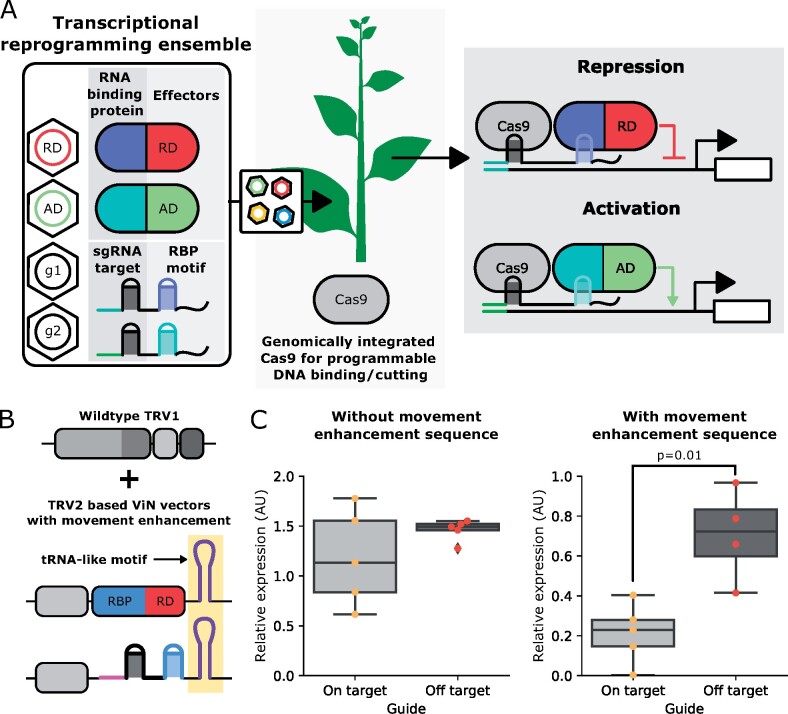
ViN 2.0 vector ensembles composed of vectors with movement enhancement sequences enable transcriptional reprograming in Cas9-expressing plants. A, Schematic describing how ViN 2.0 ensembles can be used to reprogram transcription. A ViN 2.0 ensemble consists of a combination of vectors which encode either RNA-binding protein-transcriptional effector domain fusions or sgRNA scaffolds (right inset). When these ensembles are delivered to the Cas9 line (center) the components reconstitute the desired transcription factors at the locus of interest (right). B, Schematic describing how the TRV-based vectors in a ViN 2.0 ensemble can be engineered to incorporate movement enhancement motifs (highlighted in yellow). C, Boxplots summarizing expression of *PDS1*, normalized to the expression of the *EF-1 alpha* housekeeping gene, collected from systemic leaves of Cas9-expressing *N. benthamiana* treated with ViN 2.0 repressor ensembles that encode on-target (light gray) or off-target (dark gray) sgRNA scaffolds. The vectors used in the experiments described in the plot on the left did not contain movement enhancement sequences while the vectors described in the plot on the right did. For the boxplots, the horizontal line through the box marks the median, the box is the range from the 25th to the 75th percentile, and the whiskers show the highest and lowest data points within 1.5 times the interquartile range. Each dot represents data from independent biological replicates (*n* = 4–5 per treatment). Reported *P*-values were calculated using a *t* test; *P* < 0.05 was considered significant.

While plant lines engineered to enable scaffold-based reconstitution of transcription factors enable flexible transcriptional reprogramming in a plant, this strategy is still limited to the transcriptional effector domains integrated into the genome. It has been shown that transcriptional effector domains can have variable efficacy depending on the locus being targeted (Piatek et al., 2014), which means in certain cases, it might be necessary to screen several domains to achieve the desired effect. This would require generating a new transgenic line each time. To overcome this issue, we envisaged ViN 2.0, where ensembles of ViN vectors are used to deliver a combination of sgRNA scaffolds and RNA-binding protein-transcriptional effector fusions to a transgenic plant line constitutively expressing Cas9 (Figure 4A). This would enable the reconstitution of the desired transcription factors, in a similar fashion to the Arabidopsis lines described previously; however, in this case the plant would only need to be expressing Cas9.

In contrast to the approach of using ViN vectors to deliver just sgRNA scaffolds to a line already expressing a synthetic transcription factor (ViN 1.0), in ViN 2.0, two vectors need to co-localize in the same cell to enable effective transcriptional regulation: the vector encoding the transcriptional effector and the vector encoding the sgRNA scaffold. This is challenging because TRV, like most RNA viruses, tends to move through plants in a nonuniform manner, making cellular co-localization of multiple vectors likely a relatively rare event (Macfarlane, 2010). We reasoned that co-localization might be improved by incorporating a previously characterized RNA movement enhancement motif into the ViN vectors (Figure 4B). This motif was the first 102 bp of the Arabidopsis FLOWERING LOCUS T (FT) mRNA, which adopts a tRNA-like structure (Li et al., 2011). It has been previously shown to be systemically mobile (Kehr and Kragler, 2018; Liu and Chen, 2018) and enhance viral movement (Li et al., 2011).

To validate the hypothesis that the incorporation of the movement enhancing FT motif could enable the co-localization of multiple ViN vectors in systemic tissues, we performed a somatic editing-based experiment. A Cas9-expressing transgenic line of *N. benthamiana* was co-infected with two different ViN vectors, each encoding a full-length sgRNA that targets different sites in the *PHYTOENE DESATURASE 1* (*PDS1*) gene for cleavage, either with or without the FT motif. If the two vectors are able to co-localize, we would expect to see a deletion between the two sgRNA-binding sites with some frequency. Upon evaluating editing outcomes in systemic tissues 2 weeks post infiltration, we observe a significant increase in deletions between the sgRNA target sites in plants treated with the ViN vectors that incorporated the FT motif, as compared to those that did not (Supplemental Figure S6). These results demonstrate that the FT motif can improve the co-localization of ViN vectors and how ViN vectors can be applied to implement efficient somatic gene ablation.

To validate that this enhanced co-localization improves the efficacy of regulation, we then built ViN vector ensembles that encoded a repressor, SRDX (Piatek et al., 2014), as well as sgRNA scaffolds targeting it to the *PDS1* gene in a Cas9-expressing transgenic line of *N. benthamiana*. One ensemble was built with vectors that had the movement enhancement sequence, and the other without. When we assayed *PDS1* expression in systemic leaves 3 weeks after vector delivery, we observed significant repression of *PDS1* expression compared to off-target controls in plants treated with the ensemble that included the movement enhancement sequence (Figure 4C). In contrast, the ensemble that did not contain a movement enhancement sequence did not show a significant decrease in expression of *PDS1*. This is consistent with the hypothesis that greater vector mobility improves efficacy of transcriptional regulation.

We also assayed the vector copy number within the collected tissue using RT-qPCR and observed similar levels in plants treated with ensembles with and without the movement enhancement sequences (Supplemental Figure S7). This further reinforces the idea that the improved regulation is due to an enhanced movement of the vectors rather than an increase in vector concentration due to enhanced stability conferred by the tRNA-like sequence. We also explored if targeting Cas9 to the promoter of the *PDS1* was sufficient to confer repression on its own, which would imply that co-localization of the repressor and sgRNA scaffold was not necessary. We observed that plants that were treated with ensembles that do not encode a repressor showed levels of *PDS1* expression indistinguishable from off-target controls in systemic leaves at 3 weeks post-delivery (Supplemental Figure S8). This demonstrates that the entire ensemble is necessary for effective regulation.

### ViN 2.0 enables easy swapping of effectors and targets

**Figure 5:**
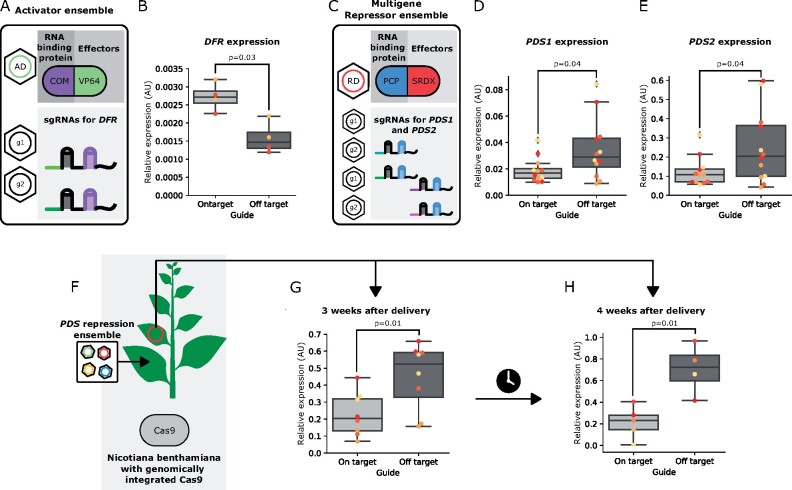
ViN 2.0 ensembles can implement multiplexed repression and activation, systemically and persistently, in a Cas9-expressing plant. A, Schematic depicting the components of the ViN 2.0 activator ensemble used to target the VP64 activator to the *DFR* gene in a *N. benthamiana* line constitutively expressing Cas9. B, Boxplots summarizing normalized *DFR* expression data from the systemic leaves of plants treated with ensembles that encode either on-target (light gray) or off-target (dark gray) sgRNA scaffolds. For all boxplots in this feature, the horizontal line through the box marks the median, the box is the range from the 25th to the 75th percentile, and the whiskers show the highest and lowest data points within 1.5 times the interquartile range. Each dot of a different color represents data collected from different leaves of independent biological replicates (*n* = 2 per treatment). Reported *P*-values were calculated using a *t* test; *P* < 0.05 was considered significant. C, Schematic depicting the components of the ViN 2.0 repressor ensemble used to target the SRDX repressor to the *PDS1* and *PDS2* genes in the same line. D and E, Boxplots summarizing *PDS1* (D) and *PDS2* (E) expression data, normalized to the expression of the *EF-1 alpha* housekeeping gene, from systemic leaves of plants treated with ensembles that encode either on-target (light gray) or off-target (dark gray) sgRNA scaffolds. Each dot of a different color represents data collected from systemic leaves of independent biological replicates (*n* = 4 per treatment). Reported *P*-values were calculated using a *t* test; *P* < 0.05 was considered significant. F, Schematic depicting a ViN 2.0 repressor ensemble targeted to the *PDS1* gene being delivered to a Cas9-expressing line of *N. benthamiana*, with the second systemic leaf highlighted with a red circle. G and H, Box plots summarizing normalized *PDS1* expression data from the second systemic leaves 3 weeks (G) and 4 weeks (H) after vector delivery from plants treated with ensembles that encode either on-target (light gray) or off-target (dark gray) sgRNA scaffolds. Each dot of a different color represents data collected from systemic leaves of independent biological replicates (*n* = 4 per treatment). Reported *P*-values were calculated using a *t* test; *P* < 0.05 was considered significant. AU, arbitrary units; COM, an RNA-binding protein.

One of the key advantages of ViN 2.0 over ViN 1.0 is the capacity to easily swap effectors by changing the vectors within an ensemble. To demonstrate this, we built an ensemble of ViN vectors, one encoding a VP64 activator and the other encoding sgRNA scaffolds to target the *DFR* gene, a metabolic gene previously described as a good target for activation (Selma et al., 2019), in a *N. benthamiana* plant stably expressing Cas9 (Figure 5A). We then collected tissue from systemic leaves 3 weeks after delivery and quantified the expression of the *DFR* gene using RT-qPCR. We observed a significant increase in the expression of *DFR* in plants treated with an ensemble that encoded on-target sgRNAs compared to plants treated with ensembles which encoded off-target control sgRNAs (Figure 5B).

Creating certain traits may require the regulation of multiple genes simultaneously due to redundancy, a common phenomenon in plants, which often have duplicated genomes. We set out to test if ViN 2.0 ensembles could be used to alter the regulation of multiple genes simultaneously. We built a new ensemble where one ViN vector encoded an SRDX repressor and the remaining vectors encoded sgRNA scaffolds that target the *PDS1* and *PDS2* genes in a Cas9-expressing *N. benthamiana* line (Figure 5C). We then collected tissue from systemic leaves 3 weeks after delivery and quantified the expression of *PDS1* and *PDS2* using RT-qPCR. We observed significant repression of expression of both *PDS1* and *PDS2* genes compared to plants treated with ensembles that encode off-target controls (Figure 5, D and E).

### Characterizing the spatio-temporal gene expression changes conferred by ViN 2.0 ensembles

An important consideration for any mobile vector is characterizing the spatio-temporal trajectory of gene expression through the plant post-delivery. We delivered ensembles targeting a repressor to the *PDS1* gene in a Cas9-expressing *N. benthamiana* line and characterized expression of *PDS1* compared to off-target controls in systemic leaves (Figure 5F). We observed significant repression of *PDS1* expression in the second systemic leaf (two leaves above the leaf delivered to) 3 weeks after delivery (Figure 5G). We characterized the expression of *PDS1* over time and observed that the repression of expression was still observed at 4 weeks post vector delivery (Figure 5H).

We also characterized vector abundance throughout the plant post-delivery and observed the expected gradient of vector concentration decreasing from the point of delivery (Macfarlane, 2010) at 2 weeks post-delivery (Supplemental Figure S9). This gradient had equalized by 4 weeks, with equivalent levels of vector in the second and fourth systemic leaves (Supplemental Figure S9). We also observed that while we do see the expected trend of decrease in median *PDS1* expression compared to control levels in the fourth and sixth systemic leaves, the difference compared to the off-target control is too small to be significant (Supplemental Figure S10). This is most likely because the baseline expression of *PDS1* in younger leaves is lower, as can be seen from the progressively lower *PDS1* expression in off-target controls in the fourth and sixth systemic leaves, while the on-target expression remains at the same maximally repressed level seen in the second systemic leaf. These results suggest that repression is being implemented systemically.

### ViN 2.0 ensembles can rapidly generate transcriptional alterations and associated phenotypic changes in tomato

**Figure 6:**
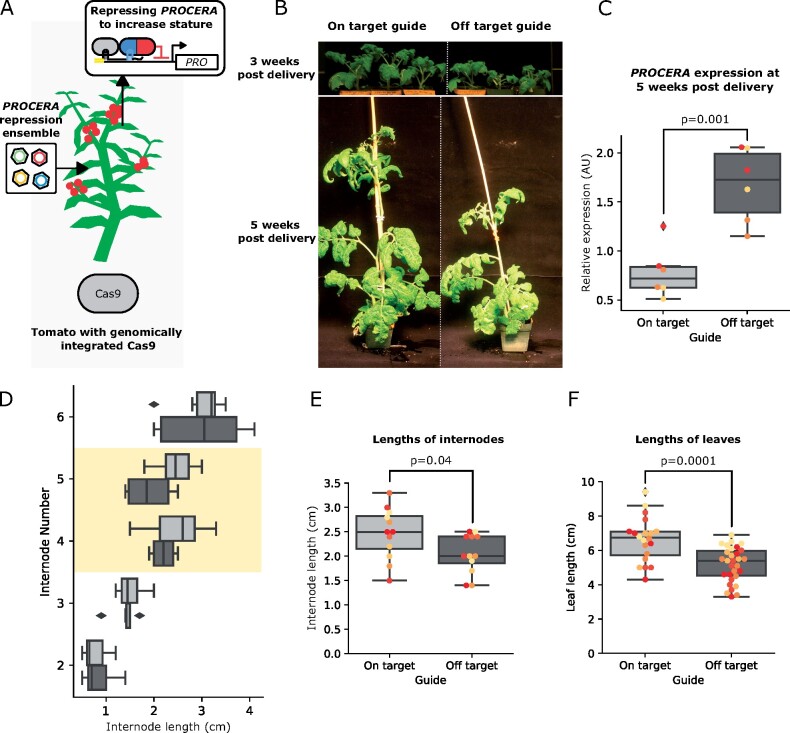
ViN 2.0 ensembles can repress *PROCERA* expression systemically and rapidly create increased stature and organ size. A, Schematic describing the ViN 2.0 repressor ensemble used to deliver a TPLN188 repressor and sgRNA scaffolds to target the *PROCERA* gene in a tomato line that stably expresses Cas9. The inset panels show the resultant reconstituted transcription factor at the *PROCERA* promoter. B, Representative images of plants treated with ensembles encoding off-target (left) and on-target (right) sgRNA scaffolds, 3 weeks (top), and 5 weeks (bottom) post vector delivery. C, Boxplot summarizing relative expression of *PROCERA*, normalized to the housekeeping gene *qCAC*, from systemic leaves of the plant lines described in (A) that were treated with ViN vectors encoding off-target (light gray) or on-target (dark gray) sgRNAs. For all boxplots in this fiture, the horizontal line through the box marks the median, the box is the range from the 25th to the 75th percentile, and the whiskers show the highest and lowest data points within 1.5 times the interquartile range. Each dot of a different color represents data from independent biological replicates (*n* = 3 per treatment). Reported *P*-values were calculated using a *t* test; *P* < 0.05 was considered significant. D, Boxplots summarizing the internode lengths of the plants described in (A) treated with ViN vectors encoding off-target (light gray) or on-target (dark gray) sgRNAs. Internode number 1 is at the bottom of the plant. The yellow region highlights the internodes in which we see a significant difference in size between plants treated with on-target and off-target sgRNAs. E, Boxplots summarizing the lengths of the fourth and fifth internodes, which are highlighted in yellow in (D), of the plants described in (A) treated with ViN vectors encoding off-target (light gray) or on-target (dark gray) sgRNAs. Each dot of a different color represents data from an independent biological replicate (*n* = 6 per treatment). Reported *P*-values were calculated using a *t* test; *P* < 0.05 was considered significant. F, Boxplots summarizing the lengths of leaves on the eight systemic branch of the plants described in (A) treated with ViN vectors encoding off-target (light gray) or on-target (dark gray) sgRNAs. Each dot of a different color represents data from an independent biological replicate (*n* = 5 per treatment). Reported *P*-values were calculated using a *t* test; *P* < 0.05 was considered significant.

Our results so far demonstrate the utility of ViN to enable rapid hypothesis testing in two model species, Arabidopsis and *N. benthamiana*. We next set out to test if these vectors could be extended to a crop plant, *S. lycopersicum* (tomato). Given our success with creating predictable morphological alterations with ViN 1.0 in Arabidopsis and *N. benthamiana* through changes to the GA signaling pathway, we explored if this approach could be applied to alter the stature of tomato. We built ensembles that encode the 188 N-terminal residues of the TOPLESS co-repressor from Arabidopsis and sgRNA scaffolds targeting the tomato DELLA protein, PROCERA (Figure 6A). This truncation has been previously shown to be sufficient to confer repression (Pierre-Jerome et al., 2014) and was necessary to reduce the size of transcriptional effector cargo within the viral cargo capacity. We decided to target *PROCERA*, as this gene has been well studied in tomato (Jupe et al., 1988; Bassel et al., 2008; Lor et al., 2014), and so we could make good predictions of the phenotypic results of altered expression. Additionally, tomato only has a single *DELLA* gene, so targeting it reduces the chance of not observing a phenotype due to genetic redundancy.

We delivered the ensemble targeting a repressor to the *PROCERA* promoter in parallel with another ensemble encoding off-target controls in Cas9-expressing tomato plants (var. M82) at the two true leaf stage and analyzed *PROCERA* expression in systemic leaves after 5 weeks. We observed a significant repression of *PROCERA* expression in plants treated with ensembles that encoded on-target sgRNAs compared to off-target controls (Figure 6C). We also observed the expected gradient of vector concentration decreasing from the point of delivery, demonstrating effective systemic movement of the vectors (Supplemental Figure S11).

Previous studies of *PROCERA* mutants have demonstrated that knocking out this gene leads to an increase in both stature and organ size (Jupe et al., 1988; Lor et al., 2014). These results and the model of GA signaling (Jupe et al., 1988; Middleton et al., 2012; Bassel et al., 2008; Jasinski et al., 2008; Carrera et al., 2012; Lor et al., 2014) imply that repressing *PROCERA* expression, rather than knocking it out, would result in a more subtle increase in stature and organ size. We phenotyped the internode length and observed a significant increase in the lengths of the fourth and fifth internodes (Figure 6, B, D and E). We hypothesize that we do not see increased length in the first few internodes, as it takes time for the vector concentration and co-localization to build to the levels that can create physiologically relevant changes in the expression of *PROCERA*. We believe that the top internode was early in its development and not fully extended, which is why we did not see a significant difference there. This mirrors the pattern of change in leaf size we report in our experiments with ViN 1.0 in *N. benthamiana* in Figure 2. This pattern was further replicated in a second trial with independently grown plants, further confirming these results were not due to growth conditions or positional effects (Supplemental Figure S12).

We also observed a significant increase in the length of systemic leaves in plants treated with ensembles encoding on-target sgRNAs compared to controls (Figure 6F). This is consistent with the repression of *DELLA* expression leading to increased tissue elongation (Band et al., 2012). These phenotypes were achieved within 5 weeks of vector delivery, compared to the months to years it would take to generate comparable phenotypes via transgenic approaches (Lor et al., 2014).

## Discussion

In this work, we demonstrate how the ViN 1.0 platform can be used to rapidly reprogram both phytohormone-mediated and static transcriptional regulation in the model plants, *A. thaliana* and *N. benthamiana*. This expression alteration is observed across the plant and persists for several weeks. All of our experiments focused on aerial vegetative tissue, specifically the leaves and stem, so more work needs to be done to explore the utility of ViN vector-based expression modulation in other tissues. We show how ViN vectors can be deployed in HACR lines, guided by a model of GA signaling (Band et al., 2012; Middleton et al., 2012), to alter the expression of key genes in this pathway and create predictable GA biosynthesis and dwarfing phenotypes. We also show how plant lines engineered to enable scaffold-based reconstitution of transcription factors allow simultaneous activation and repression of genes to tune multiple pathways and thereby stack traits, namely dwarfing and anthocyanin accumulation. These synthetic transcription factors have been previously shown capable of tuning gene expression (Piatek et al., 2014; Zalatan et al., 2015), making them powerful tools for plant engineering. Our results highlight how these tools might be used to reprogram the expression of multiple genes simultaneously to explore and engineer multigenic phenotypes.

While we do observe some variability in the strength of regulation across biological replicates, the magnitude is comparable to variability observed in previously characterized transgenic lines stably expressing synthetic transcription factors (Khakhar et al., 2018). The modest changes in gene expression we report are in contrast to the near total abrogation of gene expression that can be observed with the other predominant mode of virus-based expression perturbation, VIGs. For example, while VIGs-based knockdown of *PDS1* leads to a bleaching phenotype, characteristic of a total knockout of *PDS1* expression, ViN-based knockdown is more modest and so does not create a phenotype. This highlights that, at present, VIGs are a more appropriate tool for large fold change knockdowns of gene expression, whereas ViN is best suited to achieve modest repression. However, ViN has the added utility of enabling activation of gene expression, which is not possible via VIGs, making it a useful complementary technology. Additionally, while not explored in this work, the synthetic transcription factors deployed by ViN vectors have also been demonstrated to be capable of tuning gene expression, and it is not obvious how this could be achieved with VIGs. Finally, the incorporation of domains that respond to internal or external cues into synthetic transcription factors allows ViN vectors to implement dynamic and conditional regulation, as compared to the static repression implemented by VIGs. This expands the scope of questions that can be asked with ViN vectors, as we demonstrated by weakening the negative feedback in GA biosynthesis in *N. benthamiana* using a GA-HACR.

In the future, larger fold changes might be achieved by using different transcriptional effector domains or through recruitment of multiple effectors per sgRNA. Further expanding the existing toolbox of effectors encoded by ViN vectors to include more activators, repressors, and other enzymatic domains capable of epigenetic modifications are exciting avenues for future work. The metabolic phenotypes we created serve as a proof-of-concept of how ViN vectors could be used to tune the expression of biosynthetic enzymes, like the *GA20ox* gene family, or transcription factors that regulate entire pathways, like PAP1, to rapidly engineer plant metabolism. ViN, when paired with high-throughput phenotyping, will enable rapid screening of candidate genes at scale to elucidate the core components of poorly understood metabolic pathways in the future.

We describe how ViN could be made even more flexible by using ViN 2.0 ensembles. According to our results, the incorporation of a movement enhancement motif is essential for the efficient functioning of this system. We show that this appears to be due to enhanced co-localization of the vectors, rather than enhanced concentration. In the future, single-cell sequencing approaches could be used to further characterize the degree of co-localization of these vectors. Additionally, there are several systemically mobile tRNA-like sequences that have been reported, some with tissue-specific movement patterns (Kehr and Kragler, 2018), which represent a potential avenue to improve ViN vector performance or tissue specificity.

While ViN 2.0 does confer increased flexibility and obviates the need to build plant lines stably expressing transcription factor components, this approach does have some limitations. It can only be used to deploy relatively small transcriptional effectors due to the limited cargo capacity (<1 kb) of these vectors. Additionally, while ViN 2.0 requires co-localization of at least two vectors in the same cell for effective regulation, ViN 1.0 does not. This might increase VIN 1.0’s efficacy, although we see no major evidence of this in the experiments reported in this work. A more thorough comparison using the same sgRNAs and effectors is needed to definitively determine their relative efficacy. It should be noted that ViN vectors, even with ViN 2.0 ensembles, require an initial step of transgenesis to incorporate expression cassettes for the large protein components of the synthetic transcription factors into the plant genome. Future work in this space will be needed to further increase the cargo capacity of vectors and enable systemic delivery of all synthetic transcription factor components. Thanks to the broad host range of TRV (Macfarlane, 2010) and the fact that using ViN 2.0 only requires plants that stably express Cas9, these vectors can be rapidly and widely deployed, as Cas9 lines already exist for most major crops. This capability allowed us to deploy ViN 2.0 in a Cas9-expressing line of tomato and demonstrate systemic and persistent alteration of gene expression in the GA signaling pathway.

Previous studies of the phenotypic effects of perturbing the GA signaling pathway have focused on knockouts or overexpression of key genes (Jupe et al., 1988; Bassel et al., 2008; Jasinski et al., 2008; Carrera et al., 2012; Lor et al., 2014). These extreme cases, while mechanistically informative, do not generally represent reasonable agricultural interventions as they are often associated with pleiotropic effects. By utilizing synthetic transcription factors, we can make smaller changes and examine ranges of gene expression that might yield agriculturally beneficial phenotypes without the associated pleiotropic effects. Our observation that *PAP1* activation, which was too subtle to be quantified experimentally was able to reproducibly generate a measurable metabolic phenotype, highlights how small changes in gene expression can be phenotypically meaningful. Our results in *N. benthamiana* further validate the hypothesis that negative feedback in GA biosynthesis is an important factor controlling GA homeostasis (Middleton et al., 2012) and demonstrate how GA-HACRs can be deployed to create semi-dwarfed phenotypes by weakening the strength of this feedback loop. Our experiments in *A. thaliana* show another avenue to rapidly create a semi-dwarfed phenotype is to statically repress the expression of the GA receptors, the *GID* genes. This is consistent with what we would expect from previous studies of knockouts of these genes (Griffiths et al., 2006), as well as the model of GA signaling (Middleton et al., 2012). Using ViN 2.0 in tomato, we demonstrate that repressing the expression of the tomato DELLA protein, PROCERA, is an avenue to increase internode and leaf length. As expected, the phenotypes observed were consistent but less extreme than those observed in *PROCERA* knockout lines (Lor et al., 2014). These results show, for the first time, that synthetic transcription factors can be applied to predictably increase or decrease plant size by modulating GA biosynthesis, GA perception, or GA-dependent regulation via tuning the expression of the *GA20ox*, *GID*, or *DELLA* genes, respectively. The fact that we were able to use similar strategies across various plants points to synthetic transcription factor-mediated reprogramming being a generic strategy to engineer plant size, an agriculturally relevant trait (Eshed and Lippman, 2019). Being able to tune the GA signaling pathway has been long recognized to have broad implications for improving the yield and geographical range of a range of mainstream crops like cotton and soybean, as well as orphan crops like teff and quinoa (Eshed and Lippman, 2019).

The tomato phenotypes described were generated within a few weeks of vector delivery, compared to the over a year it would take to generate similar phenotypes via transgenic interventions (Lor et al., 2014). It was also achieved via an A*grobacterium* infiltration, which requires significantly less time and technical skill than any transgenesis protocol. In the future non-*Agrobacterium*-based nucleic acid delivery methods, such as carbon nanotubes (Demirer et al., 2019), could be explored to expand these tools to species that cannot be easily infected by *Agrobacterium*. The capacity to avoid transgenesis also enables some common issues associated with the generation of stable lines to be side stepped. For example, the generational silencing of transgenes, the requirement of strong and constitutive promoters, and the necessity to screen lines to account for variation associated with the genomic context of transgene insertion. However, ViN vectors do introduce some new sources of variation associated with viral movement, tissue tropism, and potential viral pleiotropic effects, which will have to be investigated further in future studies. Additionally, by obviating the cost and skill associated with transgenesis, these tools make plant synthetic biology more accessible to the wider plant biology community.

However, while ViN vectors are powerful new tools, there are many areas for further improvement. An important area is engineering an effective and genetically stable containment and clearing system, in case they somehow escape the contained lab conditions we use them in. One potential strategy is incorporating chemically cleavable motifs, called aptazymes (Ketzer et al., 2014), into the viral genome in conserved regions via synonymous mutations. Another is removal of structural viral proteins critical for vector transmission (Macfarlane, 2010). However, truly safe vectors will likely require multiple overlapping containment mechanisms that are independent of each other in the same vector to minimize the chances of escape. Engineering ViN vectors to be asymptomatic in all conditions is another area that requires work. While we did not see strong symptoms associated with viral infection in our experimental conditions, low temperatures (20°C–22°C) can result viral symptoms in *N. benthamiana*.

The results presented here highlight how ViN will accelerate synthetic transcription factor-mediated hypothesis testing by obviating transgenesis. The speed and scalability that ViN vectors enable will help with building predictive models of plant biology for precision forward engineering of crops in the future. ViN will also allow plant engineers to rapidly iterate through different targets and effectors to identify optimal synthetic transcription factor mediated interventions for crop improvement. We hope these tools will empower the plant science community to deliver solutions to the pressing issues facing global agriculture.

## Materials and methods

### Transgenic line generation

The transgenic GA-HACR-expressing *N. benthamiana* lines used in this article were generated by transforming the previously published GA-HACR (Khakhar et al., 2018) plasmid into wild-type *N. benthamiana*. This construct includes expression cassettes for the GA-HACR itself, which is a dCas9 protein fused to the Arabidopsis (*A. thaliana*) DELLA protein *RGA* and a truncation of the Arabidopsis repressor TOPLESS, as well as for a Venus–luciferase fusion reporter and a sgRNA that targets the GA-HACR to this reporter. The transgenesis protocol previously published (Sparkes et al., 2006) was used to generate stable transgenic lines and a highly expressing T2 line was used to perform all the experiments in this article.

The Arabidopsis transgenic lines described in this article were generated by integrating either the HACKER locus 3 or HACKER locus 20 construct into the genome via floral dip (Clough and Bent, 1998). Both HACKER loci are tDNAs that contain expression cassettes for the constitutive expression of a nuclease active Cas9 codon optimized for expression in Arabidopsis. They also both contain an expression cassette for the expression of a COM RNA-binding protein fused to a VP64 activator domain via a flexible linker. The HACKER locus 3 contains an additional expression cassette that constitutively expresses a PCP RNA-binding domain fused to a truncation of the Arabidopsis repressor TOPLESS. This polypeptide is fused, via a P2A peptide, to an MCP RNA-binding domain, which in turn is fused to an auxin degron and a truncation of TOPLESS. This allows these two RNA-binding protein effector fusions to be post-translationally separated, theoretically enabling simultaneous repression, as well as well as hormone-regulated repression. Finally, the HACKER 3 locus also encodes a Venus reporter, whereas the HACKER 20 locus encodes both a *Renilla* luciferase and a firefly luciferase reporter. These constructs were built using a two-step Goldengate assembly (Engler et al., 2008), with some parts from the previously published genome engineering kit (Cermak et al., 2017).

The Cas9-expressing lines of both *N. benthamiana* and tomato (M82) used in this work were generated previously (Čermák et al., 2015). A list of all the plasmids described is available in Supplemental Table S1 and the associated GenBank sequence files are available in the supplemental information.

### ViN vector construction

The ViN vectors described in this work were all modified versions of the TRV2 genome (Dong et al., 2007). These add an additional sub-genomic promoter and the RNA which we used to deliver downstream of the coat protein coding sequence. The ViN vectors that encode a truncated guide RNA scaffold consist of a guide RNA with a 14-bp long target sequence, the handle sequence, and the motifs, which interact specifically with either the PCP or COM RNA-binding proteins, depending on the experiment. All the sequences used for on-target and off-target guide RNA scaffolds are listed in Supplemental Table S2.

The ViN vectors used in ViN 2.0 ensembles to encode effectors contain coding sequences for either the SRDX repressor domain or the 188 N-terminal residues of TOPLESS fused to a PCP RNA-binding domain, or a VP64 activator domain fused to the COM RNA-binding domain. All the vectors, unless specified, encode the tRNA-like sequence in first 102 base pairs of the Arabidopsis FLOWERING LOCUS T transcript 3′ of the cargo as a movement enhancement sequence. All these vectors were built using a one-step Golden gate assembly.

### Agrobacterium-based vector delivery

In all experiments ViN vectors were delivered via Agrobacterium infiltration of leaves of young plants (Sparkes et al., 2006). Individual Agrobacterium (GV3101) containing the tDNAs encoding the ViN vectors or the TRV1 genome were cultured overnight in LB media with Kanamycin (50 mg·mL^−1^) and Gentamycin (50 mg·mL^−1^) selection on shaker at 220 r.p.m. and 28°C. The next day (18–20 h later), once the cultures were at confluent growth, they were spun down at 2500*g* for 10 min and washed twice with infiltration media (10-mM MgCl_2_, 10-mM 2-(N-morpholino) ethanesufonic acid (MES) at pH 5.6). For infiltrations into *N. benthamiana* these cultures were then resuspended in infiltration media at an OD_600_ of 0.6 and allowed to rest room temperature for 2–3 h prior to infiltration. For infiltrations into Arabidopsis these cultures were then resuspended in infiltration media with acetosyringone (200 μM final concentration) at an OD_600_ of 1 and allowed to rest room temperature for 2–3 h prior to infiltration. For infiltrations into tomato, these cultures were then resuspended in infiltration media with Acetosyringone (200 μM final concentration) at an OD_600_ of 2 and allowed to rest room temperature for 2–3 h prior to infiltration. For all assays performed a 1:1 ratio of by volume of Agrobacterium containing TRV1 to TRV2 was used. For ViN 1.0 the final infiltrated mixtures of Agrobacterium contained equal volumes of strains that contained the sgRNA encoding TRV2s. For ViN 2.0 the final infiltrated mixtures of Agrobacterium contained equal volumes of strains that contained the sgRNA encoding TRV2s as well as one volume of the strain that contained an RNA-binding protein–effector fusion encoding TRV2 per volume of the sgRNA encoding TRV2s.

### GA-HACR-based luciferase assay

For the luciferase assays that were used as a proxy for GA concentration with the GA-HACR *N. benthamiana* lines treated with ViN vectors, systemic leaves were assayed at the time of phenotyping, 9 weeks post vector delivery. Luciferin (100 μM final concentration in water) was infiltrated into the leaves being assayed and they were removed from the plants and imaged after 5 min. The leaves were imaged using a CCD camera-based luciferase imaging 8-min exposures of plant tissue were taken in using a UVP BioImaging Systems EpiChemi3 Darkroom with a 10-min exposure. ImageJ (Schindelin et al., 2012) was used to quantify the luminescence in each infiltrated zone with three technical replicates per measurement.

### GA dose–response characterization of GA HACR-lines

For the dose–response characterization, GA-HACR *N. benthamiana* lines treated with ViN vectors encoding either on or off target guides, and after 3 weeks the fourth systemic leaves on each plant were treated with either mock, 0.01 μM, or 100 μM GA in infiltration media (10-mM MgCl_2_, 10-mM MES at pH 5.6). Tissue was then harvested after 3 h and RNA was extracted using Trizol reagent. Expression of the *GA20ox1-3* gene was characterized from this RNA with RT-qPCR.

### Expression analysis via RT-qPCR

For all expression analysis in this work, tissue was collected from systemic tissues on plants treated with ViN vectors and RNA was extracted using the TRIZOL reagent (Invitrogen). After a DNAse treatment with the TURBO-DNAse kit (Invitrogen), the concentration of RNA of the targeted genes, the ViN vectors and housekeeping genes was quantified using RT-qPCR performed with the one-step SuperScript RT-PCR kit (Invitrogen) on a BioRAD thermocycler. Reactions were scaled down to 12.5 μL with 50 ng of RNA per reaction to conserve reagents. Between one and two technical replicates were performed on all samples. The RT-qPCR primers used are listed in Supplemental Table S3.

### Phenotyping *N. benthamiana* leaf size

The GA-HACR-expressing *N. benthamiana* plants were treated with ViN vectors and grown in a Conviron chamber set to a 18-h d (26°C) and 6-h night (24°C) light cycle. The temperature conditions were observed to be critical to prevent viral necrosis, if the temperature was dropped too low. The plants were placed in a well-mixed pattern across treatments in the chamber to prevent location specific effects confounding the results. These plants were phenotyped 9 weeks post vector delivery. The leaves on the main stem were removed and sequentially arrayed on a uniformly lit stage with a fixed camera distance and photographed. The leaf size was then calculated from these images using ImageJ (Schindelin et al., 2012). The raw images used to make measurements are included in the supplemental data.

### Analyzing deletion frequency from genome editing data

*Nicotiana benthamiana* plants expressing Cas9 were co-infected with ViN vectors that encoded two different full-length sgRNAs that target Cas9 to cleave two locations in the first exon of *PDS1* that are seventy base pairs from each other. Tissue was collected from both the infiltrated and first systemic leaf 2 weeks post-delivery and genomic DNA was extracted using the CTAB reagent (Clarke, 2009). A PCR amplicon spanning both cut sites was then analyzed using pooled next-generation sequencing. The percentage of reads that had the correct deletion between the two target sites were reported as a fraction of total reads.

### Phenotyping Arabidopsis rosette leaf size and color

The Arabidopsis plant lines were treated with ViN vectors and grown in a 18-h d and 6-h night light cycle at 22°C. These plants were phenotyped 3 weeks post vector delivery. The inflorescences were removed, and the plants were mounted on a uniformly lit stage and imaged with a Canon DSLR mounted on a fixed tripod to maintain consistent focal distance. To estimate the anthocyanin concentration in the rosette leaves, the images were then analyzed in ImageJ and the mean blue and green signals from three different rosette leaves per plant were recorded. These were then used to calculate the blue-to-green ratio, which has been established as a proxy that correlates with anthocyanin accumulation (Yang et al., 2016). To phenotype the size of the rosette leaves, the rosettes were dissected and placed sequentially on a scanner and imaged. These scans were then analyzed in ImageJ and the size of the individual leaves was recorded. The raw images that were analyzed are available in the Supplemental data.

### Quantifying anthocyanin concentration via spectrophotometry

The Arabidopsis plant lines were treated with ViN vectors and grown in a 18-h d and 6-h night light cycle at 22°C. These plants were phenotyped 3 weeks post vector delivery. Two ∼0.5-cm hole punches were taken from three healthy rosette leaves per plant, snap frozen in liquid nitrogen and then homogenized. Anthocyanin was then extracted overnight from this tissue in acidified methanol, and the concentration in a diluted extract was quantified based on absorbance at 536 nm (Duarte et al., 2013).

### Phenotyping leaf and internode length

The Cas9-expressing tomato (*S. lycopersicum*) M82 lines were treated with ViN vectors and grown in a Conviron chamber set to an 18-h d (26°C) and 6-h night (24°C) light cycle. The plants were placed in a well-mixed pattern across treatments in the chamber to prevent location specific effects confounding the results. These plants were phenotyped 50 d post viral delivery. The length of the internodes and the leaves were physically measured using a measuring tape. Images of the plants were also taken for one set of plants over the course of their growth, as presented in Figure 6.

## Data analysis and plotting

All the data analysis was performed in python and the associated jupyter notebooks are available in the supplemental data. All *P*-values reported were calculated using the *t* test function in the scipy package. All the data presented was plotted using the seaborn package^43^ in python. All the raw data and code used to analyze it are available on the following github repository (https://github.com/arjunkhakhar/Viparinama).

### Accession numbers

Accession numbers and sequence identifiers for genes referenced in the manuscript are provided in Supplemental Table S4.

## Supplemental data

The following materials are available in the online version of this article.

**Supplemental Figure S1**. Relative expression of the other *GA20ox* genes targeted in on- and off-target plants.

**Supplemental Figure S2**. Phenotype alterations created by ViN 1.0 in *N. benthamiana* GA-HACR lines can be replicated in a second set of independently grown plants.

**Supplemental Figure S3**. Targeting a HACR to regulate the expression of *GA20ox* reduces strength of negative feedback in GA expression.

**Supplemental Figure S4**. *PAP1*-associated anthocyanin phenotype can be obtained independently of *GID* repression.

**Supplemental Figure S5**. Activation of *PAP1* leads to increased anthocyanin accumulation.

**Supplementary Figure S6**. Incorporation of FT-motif into ViN vectors enhances co-localization of vectors in an ensemble.

**Supplemental Figure S7**. Movement enhancement motif does not confer enhanced stability to the ViN vectors.

**Supplemental Figure S8**. ViN 2.0-based repression requires the presence of the repressor domain.

**Supplemental Figure S9**. Spatio-temporal quantification of relative ViN vector abundance in the plant.

**Supplemental Figure S10**. Quantification of *PDS1* repression in tissue progressively distal from the point of infiltration.

**Supplemental Figure S11**. ViN vector abundance decreases in progressively distal tissues.

**Supplemental Figure S12**. Phenotypic effects of ViN 2.0 ensembles repressing *PROCERA* can be replicated in a second set of independently grown plants.

**Supplemental Table S1**. List of plasmids.

**Supplemental Table S2**. List of sequences targeted by sgRNAs.

**Supplemental Table S3**. List of RT-qPCR primers.

**Supplemental Table S4**. Gene accessions referenced in the manuscript.

## Supplementary Material

kiab197_Supplementary_Data

## Notes

A.K. conceived the technologies, performed and supervised experiments, analyzed the data, and wrote the paper. R.S., C.W., S.S., F.R., S.S., J.H. performed experiments. D.F.V. edited the paper and procured funding for the research.

The author responsible for distribution of materials integral to the findings presented in this article in accordance with the policy described in the Instructions for Authors (https://academic.oup.com/plphys/pages/general-instructions) is: Daniel F. Voytas (voytas@umn.edu).

## References

[kiab197-B1] Achard P , Gusti A, Cheminant S, Alioua M, Dhondt S, Coppens F, Beemster GTS, Genschik P (2009) Gibberellin signaling controls cell proliferation rate in Arabidopsis. Curr Biol 19**:** 1188–1193 19576768 10.1016/j.cub.2009.05.059

[kiab197-B2] Altpeter F , Springer NM, Bartley LE, Blechl AE, Brutnell TP, Citovsky V, Conrad LJ, Gelvin SB, Jackson DP, Kausch AP, et al (2016) Advancing crop transformation in the era of genome editing. Plant Cell 28**:** 1510–1520 27335450 10.1105/tpc.16.00196 PMC4981132

[kiab197-B3] Band LR , Úbeda-Tomás S, Dyson RJ, Middleton AM, Hodgman TC, Owen MR, Jensen OE, Bennett MJ, King JR (2012) Growth-induced hormone dilution can explain the dynamics of plant root cell elongation. Proc Natl Acad Sci U S A 109: 7577–7582 22523244 10.1073/pnas.1113632109 PMC3358831

[kiab197-B4] Bassel GW , Mullen RT, Bewley JD (2008) *PROCERA* is a putative *DELLA* mutant in tomato (*Solanum lycopersicum*): effects on the seed and vegetative plant. J Exp Bot 59: 585–593 18250077 10.1093/jxb/erm354

[kiab197-B5] Carrera E , Ruiz-Rivero O, Peres LEP, Atares A, Garcia-Martinez JL (2012) Characterization of the *PROCERA* tomato mutant shows novel functions of the SlDELLA protein in the control of flower morphology, cell division and expansion, and the auxin-signaling pathway during fruit-set and development. Plant Physiol 160**:** 1581–1596 22942390 10.1104/pp.112.204552 PMC3490602

[kiab197-B6] Čermák T , Baltes NJ, Čegan R, Zhang Y, Voytas DF (2015) High-frequency, precise modification of the tomato genome. Genome Biol 16**:** 232 26541286 10.1186/s13059-015-0796-9 PMC4635538

[kiab197-B7] Cermak T , Curtin SJ, Gil-Humanes J, Čegan R, Kono TJY, Konečná E, Belanto JJ, Starker CG, Mathre JW, Greenstein RL, et al (2017) A multi-purpose toolkit to enable advanced genome engineering in plants. Plant Cell 29: 1196–1217 28522548 10.1105/tpc.16.00922 PMC5502448

[kiab197-B8] Clarke JD (2009) Cetyltrimethyl ammonium bromide (CTAB) DNA miniprep for plant DNA isolation. Cold Spring Harb Protoc 3**:** pdb-prot5177 10.1101/pdb.prot5177 20147112

[kiab197-B9] Clough SJ , Bent AF (1998) Floral dip: a simplified method for Agrobacterium‐mediated transformation of *Arabidopsis thaliana*. Plant J 16**:** 735–743 10069079 10.1046/j.1365-313x.1998.00343.x

[kiab197-B10] Cody WB , Scholthof HB (2019) Plant virus vectors 3.0: transitioning into synthetic genomics. Annu Rev Phytopathol 57**:** 1–20 31185187 10.1146/annurev-phyto-082718-100301

[kiab197-B11] Cubillos FA , Coustham V, Loudet O (2012) Lessons from eQTL mapping studies: non-coding regions and their role behind natural phenotypic variation in plants. Curr Opin Plant Biol 15**:** 192–198 22265229 10.1016/j.pbi.2012.01.005

[kiab197-B12] Demirer GS , Zhang H, Goh NS, González-Grandío E, Landry MP (2019) Carbon nanotube–mediated DNA delivery without transgene integration in intact plants. Nat Protoc 14**:** 2954–2971 31534231 10.1038/s41596-019-0208-9 PMC10496593

[kiab197-B13] Dong Y , Burch-Smith TM, Liu Y, Mamillapalli P, Dinesh-Kumar SP (2007) A ligation-independent cloning Tobacco Rattle Virus vector for high-throughput virus-induced gene silencing identifies roles for *NbMADS4-1* and -2 in floral development. Plant Physiol 145**:** 1161–1170 17932306 10.1104/pp.107.107391 PMC2151726

[kiab197-B14] Duarte B , Santos D, Marques JC, Caçador I (2013) Ecophysiological adaptations of two halophytes to salt stress: photosynthesis, PS II photochemistry and anti-oxidant feedback – implications for resilience in climate change. Plant Physiol Bioch 67**:** 178–188 10.1016/j.plaphy.2013.03.004 23579080

[kiab197-B15] Engler C , Kandzia R, Marillonnet S (2008) A one pot, one step, precision cloning method with high throughput capability. PLos One 3**:** e3647 18985154 10.1371/journal.pone.0003647 PMC2574415

[kiab197-B16] Eshed Y , Lippman ZB (2019) Revolutions in agriculture chart a course for targeted breeding of old and new crops. Science NY 366**:** eaax0025 10.1126/science.aax0025 31488704

[kiab197-B17] Fanzo J , Davis C, McLaren R, Choufani J (2018) The effect of climate change across food systems: implications for nutrition outcomes. Global Food Secur 18**:** 12–19

[kiab197-B18] Griffiths J , Murase K, Rieu I, Zentella R, Zhang Z-L, Powers SJ, Gong F, Phillips AL, Hedden P, Sun T, et al (2006) Genetic characterization and functional analysis of the GID1 gibberellin receptors in Arabidopsis. Plant Cell 18**:** 3399–3414 17194763 10.1105/tpc.106.047415 PMC1785415

[kiab197-B19] Jasinski S , Tattersall A, Piazza P, Hay A, Martinez-Garcia JF, Schmitz G, Theres K, McCormick S, Tsiantis M (2008) *PROCERA* encodes a DELLA protein that mediates control of dissected leaf form in tomato. Plant J Cell Mol Biol 56**:** 603–612 10.1111/j.1365-313X.2008.03628.x 18643984

[kiab197-B20] Jupe SC , Causton DR, Scott IM (1988) Cellular basis of the effects of gibberellin and the *PRO* gene on stem growth in tomato. Planta 174**:** 106–111 24221425 10.1007/BF00394881

[kiab197-B21] Kehr J , Kragler F (2018) Long distance RNA movement. New Phytol 218**:** 29–40 29418002 10.1111/nph.15025

[kiab197-B22] Ketzer P , Kaufmann JK, Engelhardt S, Bossow S, von Kalle C, Hartig JS, Ungerechts G, Nettelbeck DM (2014) Artificial riboswitches for gene expression and replication control of DNA and RNA viruses. Proc Natl Acad Sci U S A 111**:** E554–E562 24449891 10.1073/pnas.1318563111 PMC3918795

[kiab197-B23] Khakhar A , Leydon AR, Lemmex AC, Klavins E, Nemhauser JL (2018) Synthetic hormone-responsive transcription factors can monitor and re-program plant development. eLife 7**:** e34702 29714687 10.7554/eLife.34702 PMC5976440

[kiab197-B24] Kiani S , Chavez A, Tuttle M, Hall RN, Chari R, Ter-Ovanesyan D, Qian J, Pruitt BW, Beal J, Vora S, et al (2015) Cas9 gRNA engineering for genome editing, activation and repression. Nat Methods 12: 1051–1054 26344044 10.1038/nmeth.3580 PMC4666719

[kiab197-B25] Larsen JS , Curtis WR (2012) RNA viral vectors for improved Agrobacterium-mediated transient expression of heterologous proteins in *Nicotiana benthamiana* cell suspensions and hairy roots. BMC Biotechnol 12**:** 21 22559055 10.1186/1472-6750-12-21 PMC3403893

[kiab197-B26] Lee WJ , Jeong CY, Kwon J, Kien VV, Lee D, Hong S-W, Lee H (2016) Drastic anthocyanin increase in response to *PAP1* overexpression in *fls1* knockout mutant confers enhanced osmotic stress tolerance in *Arabidopsis thaliana*. Plant Cell Rep 35**:** 2369–2379 27562381 10.1007/s00299-016-2040-9

[kiab197-B27] Li C , Gu M, Shi N, Zhang H, Yang X, Osman T, Liu Y, Wang H, Vatish M, Jackson S, et al (2011) Mobile FT mRNA contributes to the systemic florigen signaling in floral induction. Sci Rep-UK 1**:** 73 10.1038/srep00073 PMC3216560 22355592

[kiab197-B28] Liu L , Chen X (2018) Intercellular and systemic trafficking of RNAs in plants. Nat Plants 4**:** 869–878 30390090 10.1038/s41477-018-0288-5 PMC7155933

[kiab197-B29] Lor VS , Starker CG, Voytas DF, Weiss D, Olszewski NE (2014) Targeted mutagenesis of the tomato *PROCERA* gene using transcription activator-like effector nucleases. Plant Physiol 166**:** 1288–1291 25217528 10.1104/pp.114.247593 PMC4226374

[kiab197-B30] Macfarlane SA (2010) Tobraviruses—plant pathogens and tools for biotechnology. Mol Plant Pathol 11**:** 577–583 20618713 10.1111/j.1364-3703.2010.00617.x PMC6640422

[kiab197-B31] Mahas A , Ali Z, Tashkandi M, Mahfouz MM (2019) Virus-mediated genome editing in plants using the CRISPR/Cas9 system. Methods Mol Biology Clifton (N J) 1917**:** 311–326 10.1007/978-1-4939-8991-1_23 30610646

[kiab197-B32] Middleton AM , Úbeda-Tomás S, Griffiths J, Holman T, Hedden P, Thomas SG, Phillips AL, Holdsworth MJ, Bennett MJ, King JR, et al (2012) Mathematical modeling elucidates the role of transcriptional feedback in gibberellin signaling. Proc Natl Acad Sci U S A 109**:** 7571–7576 22523240 10.1073/pnas.1113666109 PMC3358864

[kiab197-B33] Pasin F , Menzel W, Daròs J (2019) Harnessed viruses in the age of metagenomics and synthetic biology: an update on infectious clone assembly and biotechnologies of plant viruses. Plant Biotechnol J 17: 1010–1026 30677208 10.1111/pbi.13084 PMC6523588

[kiab197-B34] Piatek A , Ali Z, Baazim H, Li L, Abulfaraj A, Al‐Shareef S, Aouida M, Mahfouz MM (2014) RNA‐guided transcriptional regulation in planta via synthetic dCas9‐based transcription factors. Plant Biotechnol J 13: 578–589 25400128 10.1111/pbi.12284

[kiab197-B35] Pierre-Jerome E , Jang SS, Havens KA, Nemhauser JL, Klavins E (2014) Recapitulation of the forward nuclear auxin response pathway in yeast. Proc Natl Acad Sci U S A 111: 9407–9412 24979769 10.1073/pnas.1324147111 PMC4084466

[kiab197-B36] Schindelin J , Arganda-Carreras I, Frise E, Kaynig V, Longair M, Pietzsch T, Preibisch S, Rueden C, Saalfeld S, Schmid B, et al (2012) Fiji: an open-source platform for biological-image analysis. Nat Methods 9**:** 676–682 22743772 10.1038/nmeth.2019 PMC3855844

[kiab197-B37] Selma S , Bernabé‐Orts JM, Vazquez‐Vilar M, Diego‐Martin B, Ajenjo M, Garcia‐Carpintero V, Granell A, Orzaez D (2019) Strong gene activation in plants with genome‐wide specificity using a new orthogonal CRISPR/Cas9‐based Programmable Transcriptional Activator. Plant Biotechnol J 17**:** 1703 31034138 10.1111/pbi.13138 PMC6686126

[kiab197-B38] Serrano-Mislata A , Bencivenga S, Bush M, Schiessl K, Boden S, Sablowski R (2017) *DELLA* genes restrict inflorescence meristem function independently of plant height. Nat Plants 3**:** 749 28827519 10.1038/s41477-017-0003-y PMC5669458

[kiab197-B39] Soares JC , Santos CS, Carvalho SMP, Pintado MM, Vasconcelos MW (2019) Preserving the nutritional quality of crop plants under a changing climate: importance and strategies. Plant Soil 443**:** 1–26

[kiab197-B40] Sparkes IA , Runions J, Kearns A, Hawes C (2006) Rapid, transient expression of fluorescent fusion proteins in tobacco plants and generation of stably transformed plants. Nat Protoc 1**:** 2019–2025 17487191 10.1038/nprot.2006.286

[kiab197-B41] Velásquez AC , Chakravarthy S, Martin GB (2009) Virus-induced gene silencing (VIGS) in *Nicotiana benthamiana* and tomato. JoVE 28**:** e1292 10.3791/1292 PMC2795700 19516240

[kiab197-B42] Walther W , Stein U (2000) Viral vectors for gene transfer. Drugs 60**:** 249–271 10983732 10.2165/00003495-200060020-00002

[kiab197-B43] Weigel D , Ahn JH, Blázquez MA, Borevitz JO, Christensen SK, Fankhauser C, Ferrándiz C, Kardailsky I, Malancharuvil EJ, Neff MM, et al (2000) Activation tagging in Arabidopsis. Plant Physiol 122**:** 1003–1014 10759496 10.1104/pp.122.4.1003 PMC1539247

[kiab197-B44] Xiao J , Li H, Zhang J, Chen R, Zhang Y, Ouyang B, Wang T, Ye Z (2006) Dissection of *GA 20-oxidase* members affecting tomato morphology by RNAi-mediated silencing. Plant Growth Regul 50**:** 179–189

[kiab197-B45] Yang X , Zhang J, Guo D, Xiong X, Chang L, Niu Q, Huang D (2016) Measuring and evaluating anthocyanin in lettuce leaf based on color information. IFAC-PapersOnLine 49**:** 96–99

[kiab197-B46] Zalatan J , Lee M, Almeida R, Gilbert L (2015) Engineering complex synthetic transcriptional programs with CRISPR RNA scaffolds. Cell 160**:** 339–350 25533786 10.1016/j.cell.2014.11.052 PMC4297522

