# Supplementary material for: VipariNama: RNA viral vectors to rapidly elucidate the relationship between gene expression and phenotype
Source: Plant Physiol. 2021 May 2;186(4):2222–38. doi: 10.1093/plphys/kiab197 (PMC8331131; doi:10.1093/plphys/kiab197)
Supplement: kiab197_Supplementary_Data [file kiab197_supplementary_data.pdf]

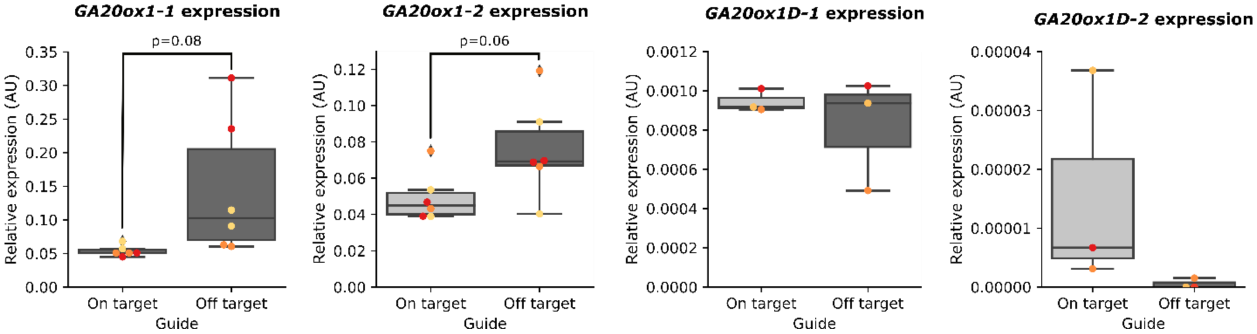

Supplemental Figure S1. Relative expression of the other GA20ox genes targeted in on- and off-target plants. Box plots summarizing expression of *GA20ox1-1, GA20ox1-2, GA20ox1D-1,* and *GA20ox1D-2*, normalized to the *EF-1 alpha* housekeeping gene, from systemic tissue of plant lines that were treated with ViN vectors encoding on-target (light grey) or off-target (dark grey) sgRNAs. In the boxplots the horizontal line through the box marks the median, the box is the range from the 25th to the 75th percentile, and the whiskers show the highest and lowest data points within 1.5 times the interquartile range. Each dot of the same color represents data from independent biological replicates (n=3 per treatment). Reported p-values were calculated using a t-test, and p<0.05 was considered significant.

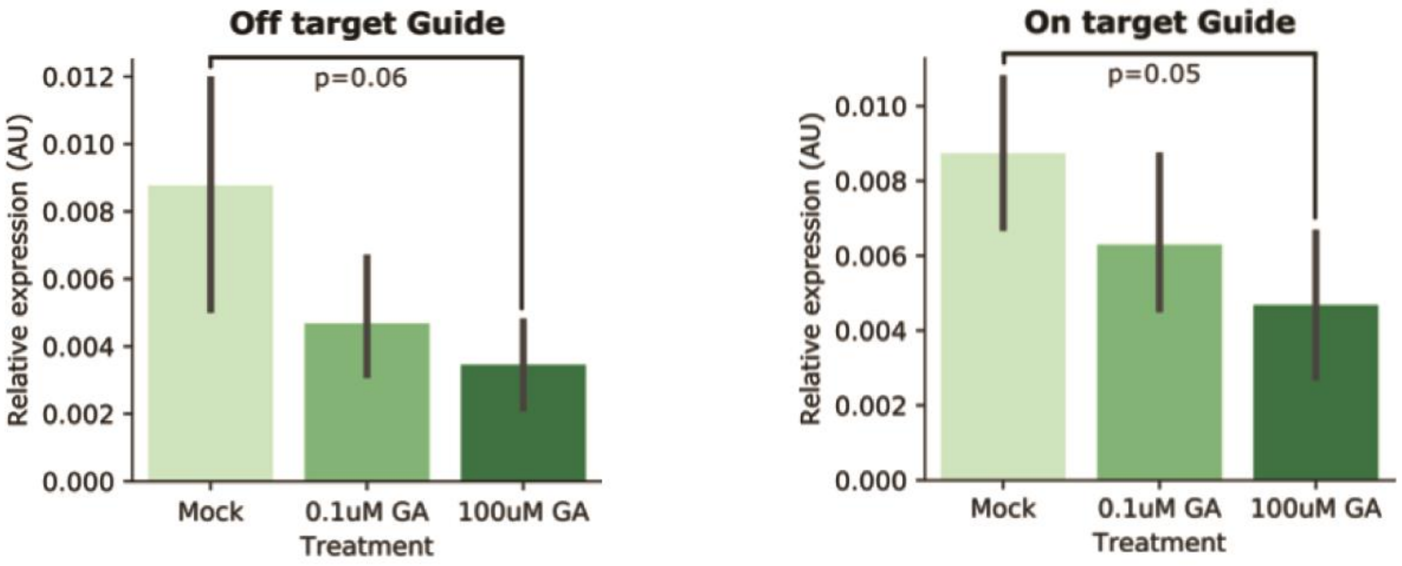

Supplemental Figure S2. Targeting a hormone activated Cas9-based repressor (HACR) to regulate expression of *GA20ox* reduces strength of negative feedback in gibberellic acid (GA) expression. Bar plots summarizing expression of *GA20ox1-3*, normalized to the *EF-1 alpha* housekeeping gene, three hours after treatment with GA from systemic tissue of the plant lines described in panel A of Figure 2 that were previously infected with ViN vectors encoding off-target (top) or on-target (bottom) sgRNAs. Each bar represents the average of four independent biological replicates, and the black lines depict standard deviation. Reported p-values were calculated using a t-test, and $p<0.05$ was considered significant.

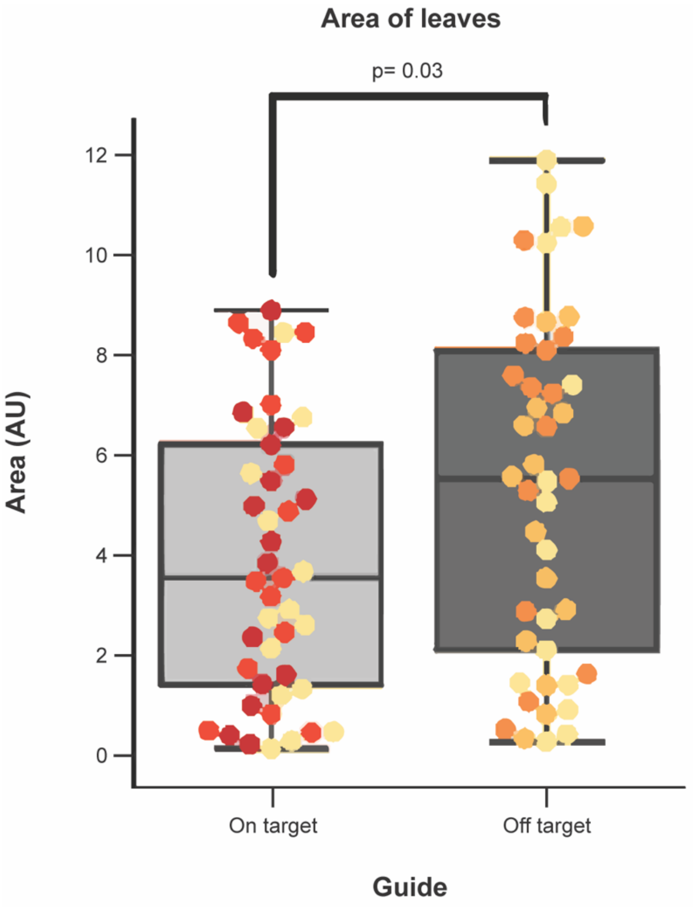

Supplemental Figure S3. Phenotype alterations created by ViN 1.0 in *N. benthamiana* GA-HACR lines can be replicated in a second set of independently grown plants. Box plots summarizing the area of all the leaves from plants treated with ViN vectors encoding on-target (light grey) or off-target (dark grey) sgRNAs. AU, arbitrary units. In the boxplots the horizontal line through the box marks the median, the box is the range from the 25th to the 75th percentile, and the whiskers show the highest and lowest data points within 1.5 times the interquartile range. Each dot of the same color represents data from a leaf of an independent biological replicate (n=3 per treatment). Reported p-values were calculated using a t-test, and $p<0.05$ was considered significant.

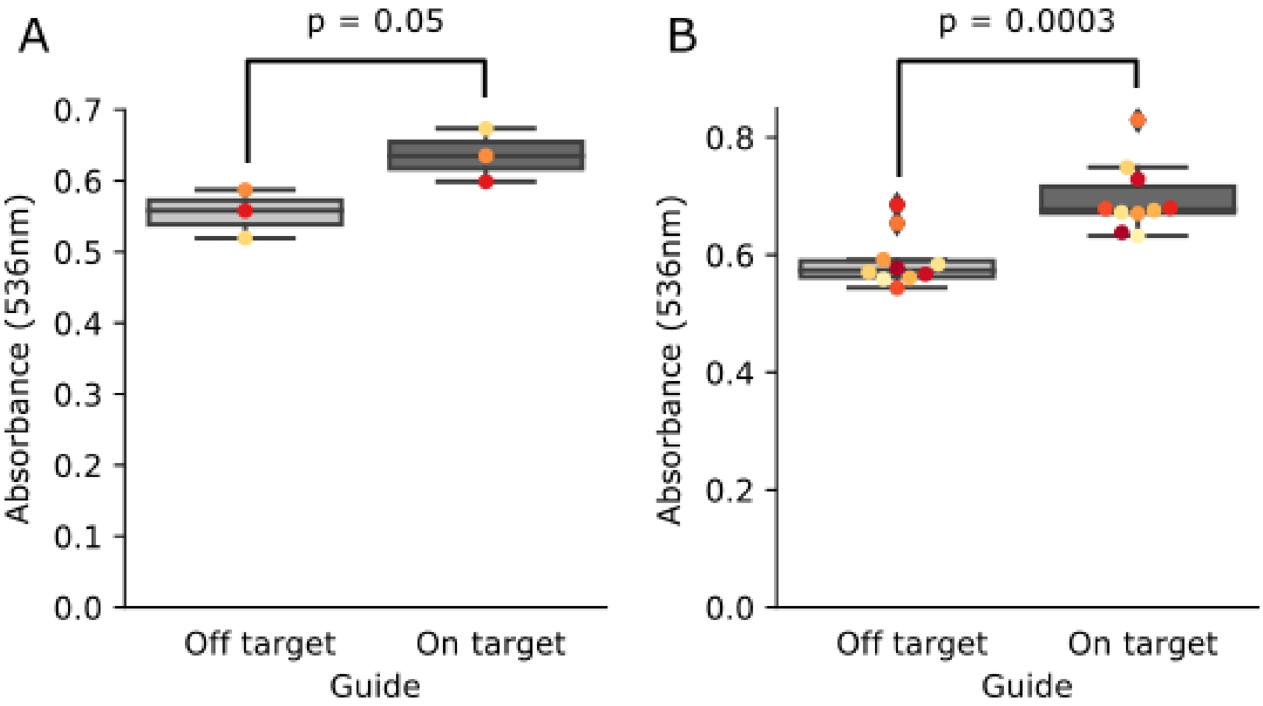

Supplemental Figure S4. Activation of *PAP1* leads to increased anthocyanin accumulation. A,B) Box plots summarizing absorbance at 536nm, the absorbance maximum of anthocyanin, from an overnight extraction in acidified methanol from systemic leaves of Arabidopsis plants described in either Figure 3A or Supplemental Figure S5 several weeks post treatment with ViN vectors encoding either on target (dark grey) or off target (light grey) guide RNAs. The horizontal line through the box marks the median, the box is the range from the 25th to the 75th percentile, and the whiskers show the highest and lowest data points within 1.5 times the interquartile range. Every colored dot represents an independent biological replicate. Reported p-values were calculated using a t-test, and $p<0.05$ was considered significant.

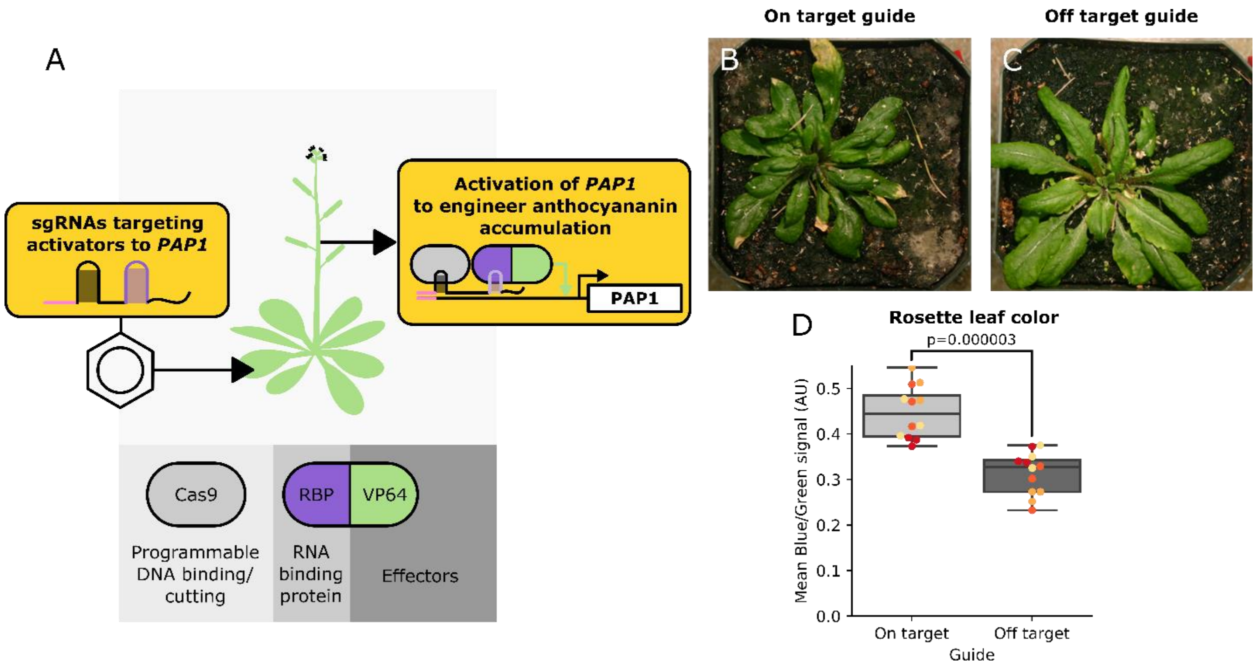

Supplemental Figure S5. ***PAP1*****-associated anthocyanin phenotype can be obtained independently of** ***GID*** **repression.** A) Schematic describing an Arabidopsis line engineered to enable scaffold-based reconstitution of transcription factors. This line constitutively expresses nuclease active Cas9 as well as an RNA binding protein fused to the VP64 activator. The colored insets describe the sgRNA scaffolds being delivered to this line via ViN vectors (left) and the resultant transcriptional perturbation as well as the expected phenotypic result (right). B,C) Representative pictures of rosettes of plants treated with on-target (B) or off-target (C) sgRNAs at the time of phenotyping. D) Box plot summarizing the average blue signal normalized to the green signal, which is an established proxy for anthocyanin concentration(Yang et al., 2016), from images of rosette leaves of the plants described in panel A that were treated with ViN vectors encoding on-target (light grey) or off-target (dark grey) sgRNAs. In the boxplots the horizontal line through the box marks the median, the box is the range from the 25th to the 75th percentile, and the whiskers show the highest and lowest data points within 1.5 times the interquartile range. Each dot of the same color represents data from leaves of independent biological replicates (n=3 per treatment). Reported p-values were calculated using a t-test, and $p<0.05$ was considered significant.

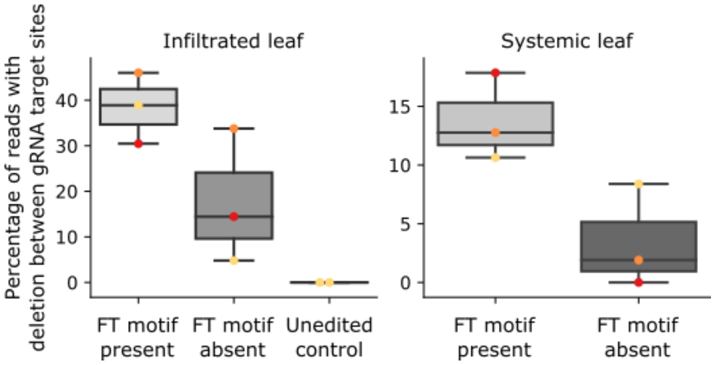

Supplementary Figure S6. Incorporation of FT-motif into ViN vectors enhances co-localization of vectors in an ensemble. Box plots summarizing frequency of deletions between target sites in *PDS1* in next generation sequencing reads of PCR amplicons of genomic DNA from Cas9-expressing *N. benthamiana* treated with ViN vector ensembles encoding full length guides that target different sites in *PDS1* that either have, or do not have, an FT motif. The plot on the left summarizes editing data from tissue collected two weeks post-delivery from the leaf to which the ViN vectors were delivered, and the plot on the right summarizes editing data from the first systemic leaf. Each dot represents an independent biological replicate. In the boxplots the horizontal line through the box marks the median, the box is the range from the 25th to the 75th percentile, and the whiskers show the highest and lowest data points within 1.5 times the interquartile range.

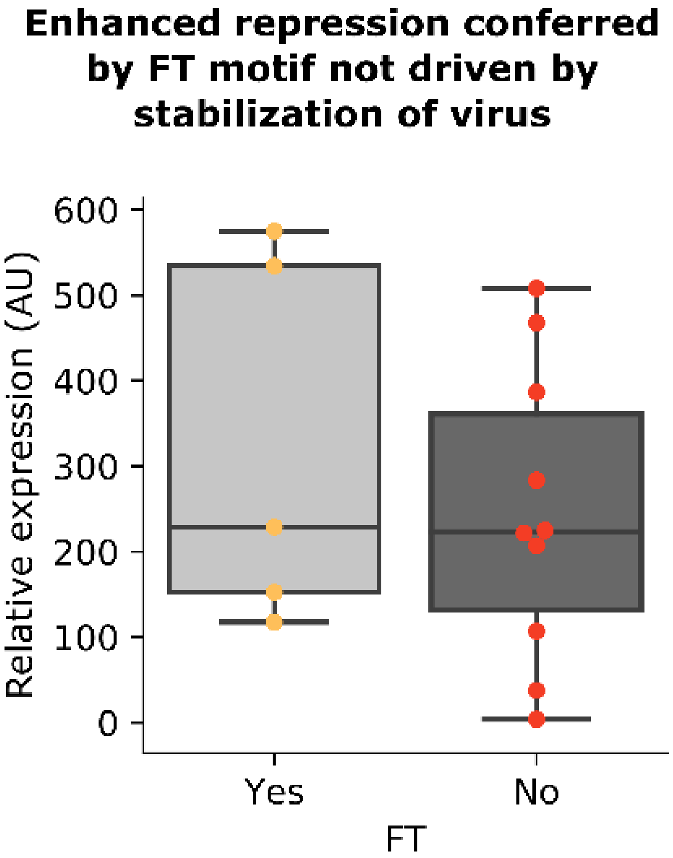

Supplemental Figure S7. Movement enhancement motif does not confer enhanced stability to the ViN vectors. Boxplots summarizing levels of the ViN vector transcripts, normalized to the expression of the *EF-1 alpha* housekeeping gene, collected from systemic leaves of Cas9-expressing *N. benthamiana* treated with ViN 2.0 repressor ensembles that either had (light grey) or did not have (dark grey) the tRNA-like motif from the Arabidopsis FT transcript. The horizontal line through the box marks the median, the box is the range from the 25th to the 75th percentile, and the whiskers show the highest and lowest data points within 1.5 times the interquartile range. Each dot represents data from independent biological replicates (n=5-10 per treatment).

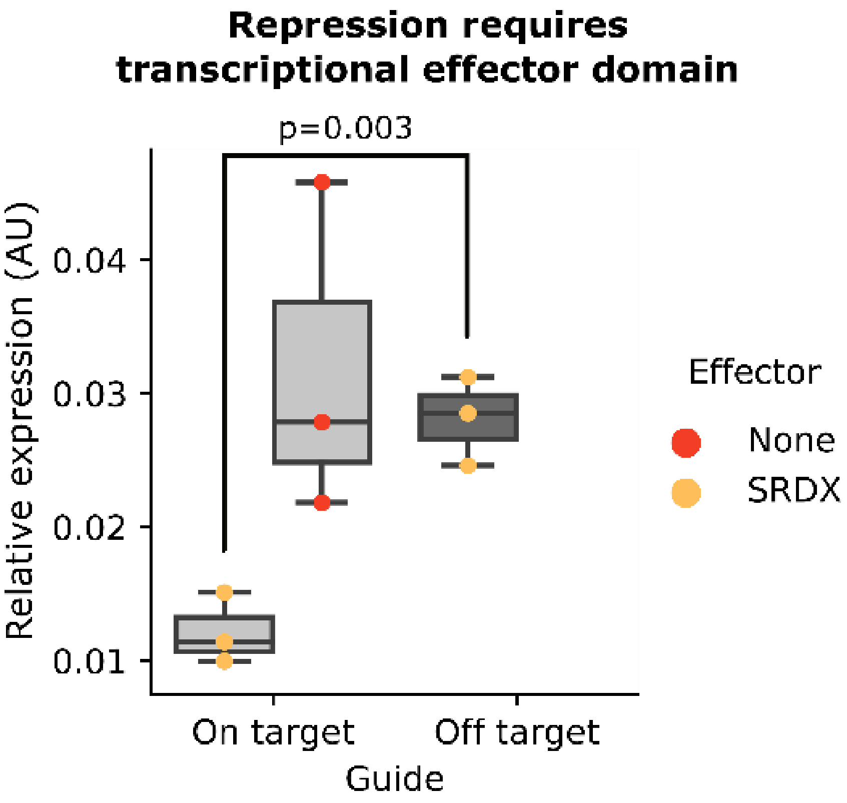

Supplemental Figure S8. ViN 2.0 based repression requires the presence of the repressor domain. Boxplots summarizing expression of *PDS1*, normalized to the expression of the housekeeping gene *EF-1 alpha*, collected from systemic leaves of Cas9-expressing *N. benthamiana* treated with ViN 2.0 ensembles that encode on-target (light grey) or off-target (dark grey) sgRNA scaffolds. The red dots represent measurements from plants treated with ensembles that did not encode a repressor, and the yellow dots from plants treated with ensembles that encoded an SRDX repressor. In the boxplots the horizontal line through the box marks the median, the box is the range from the 25th to the 75th percentile, and the whiskers show the highest and lowest data points within 1.5 times the interquartile range. Each dot represents data from independent biological replicates (n=3 per treatment). Reported p-values were calculated using a t-test, and p<0.05 was considered significant.

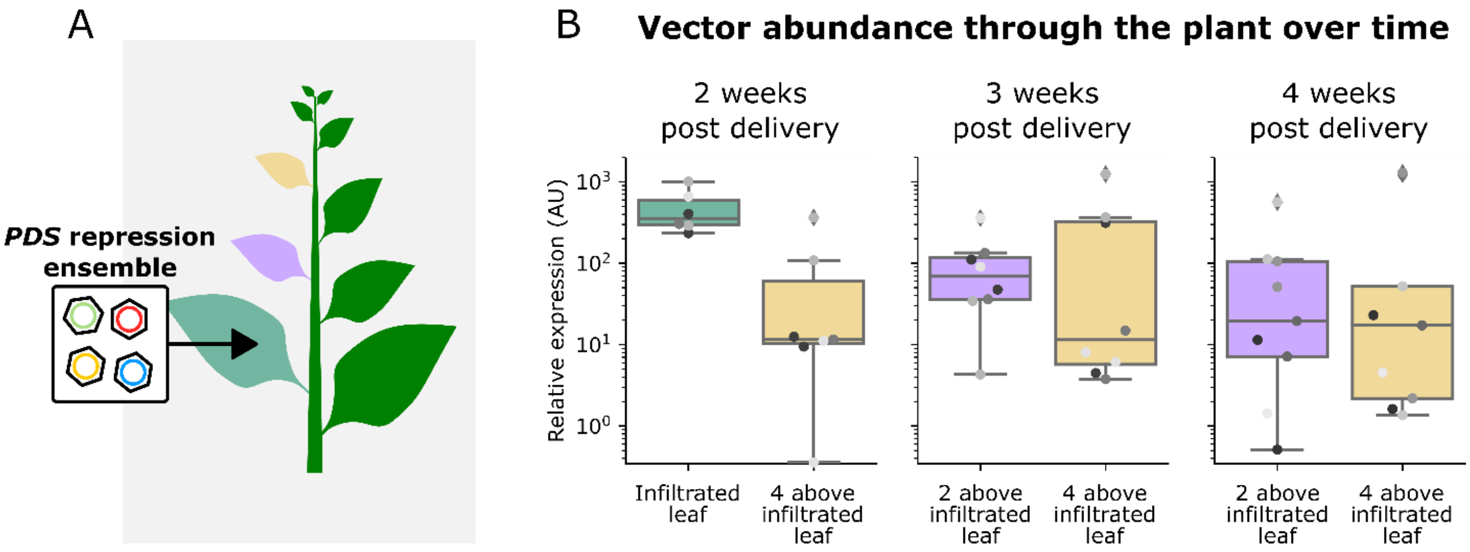

Supplemental Figure S9. Spatio-temporal quantification of relative ViN vector abundance in the plant. A) Schematic depicting a ViN 2.0 repressor ensemble targeted to the *PDS1* gene being delivered to a Cas9-expressing line of *N. benthamiana*, with different systemic leaves highlighted in non-green colors. B) Box plots summarizing normalized ViN vector RNA levels across the plant at 2 weeks, 3 weeks, and 4 weeks post vector delivery. Each boxplot is colored to match the color of the leaf it was harvested from in panel A. The horizontal line through the box marks the median, the box is the range from the 25th to the 75th percentile, and the whiskers show the highest and lowest data points within 1.5 times the interquartile range. Each dot represents data collected from systemic leaves of independent biological replicates.

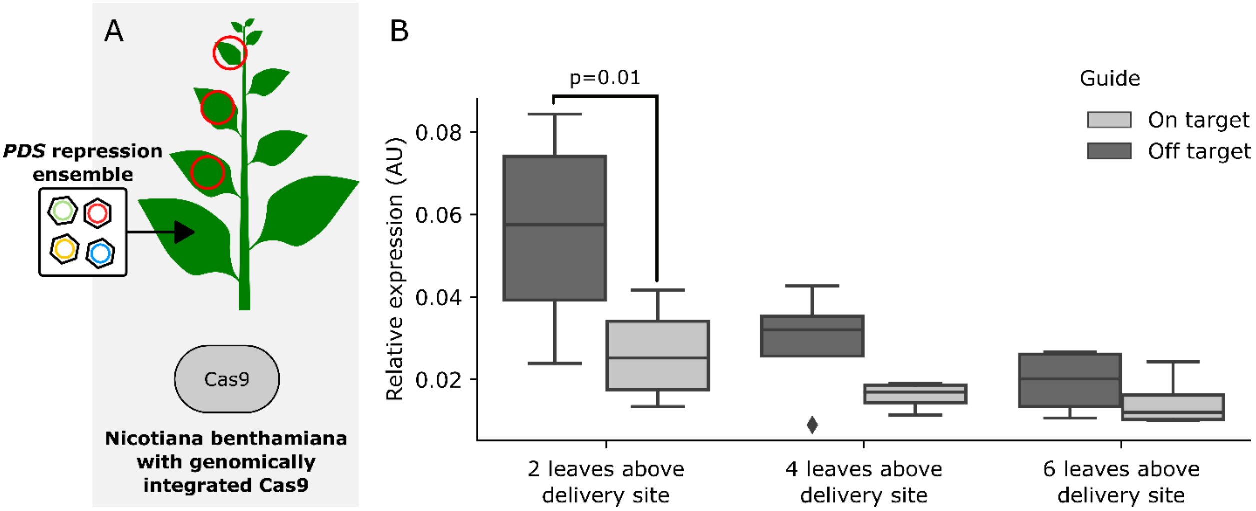

Supplemental Figure S10. Quantification of *PDS1* repression in tissue progressively distal from the point of infiltration. A) Schematic depicting a ViN 2.0 repressor ensemble targeted to the *PDS1* gene being delivered to a Cas9-expressing line of *N. benthamiana*, with the 2nd, 4th, and 6th systemic leaves highlighted with a red circle. B) Box plots summarizing normalized *PDS1* expression data, relative to the expression of the *EF-1 alpha* housekeeping gene, from systemic leaves highlighted in A after 3 weeks post treatment with ensembles that encode either on-target (light grey) or off-target (dark grey) sgRNA scaffolds. Each plot represents data collected from systemic leaves of independent biological replicates (n = 4 per treatment). The horizontal line through the box marks the median, the box is the range from the 25th to the 75th percentile, and the whiskers show the highest and lowest data points within 1.5 times the interquartile range. Reported p-values were calculated using a t-test, and $p<0.05$ was considered significant.

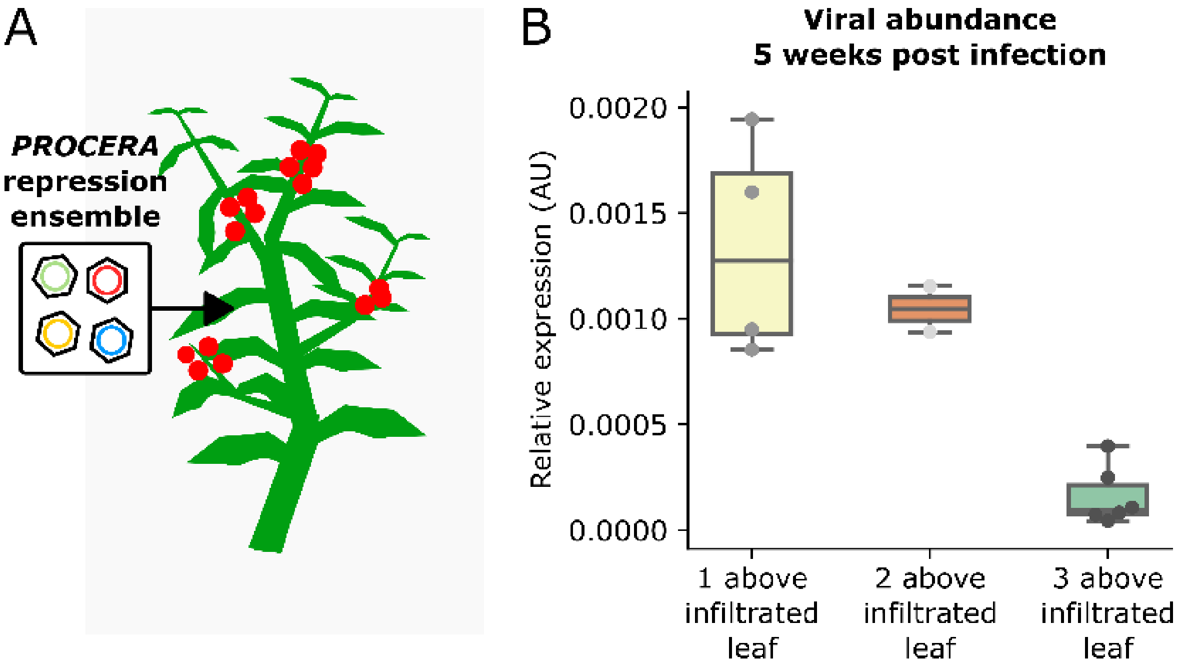

Supplemental Figure S11. ViN vector abundance decreases in progressively distal tissues. A) Schematic describing the ViN 2.0 repressor ensemble used to deliver a TPLN188 repressor and sgRNA scaffolds to target the *PROCERA* gene in a tomato line that stably expresses Cas9. B) Boxplot summarizing normalized levels of RNA of ViN vectors from systemic leaves of the plant lines described in panel A. The horizontal line through the box marks the median, the box is the range from the 25th to the 75th percentile, and the whiskers show the highest and lowest data points within 1.5 times the interquartile range. Each dot represents data from independent biological replicates.

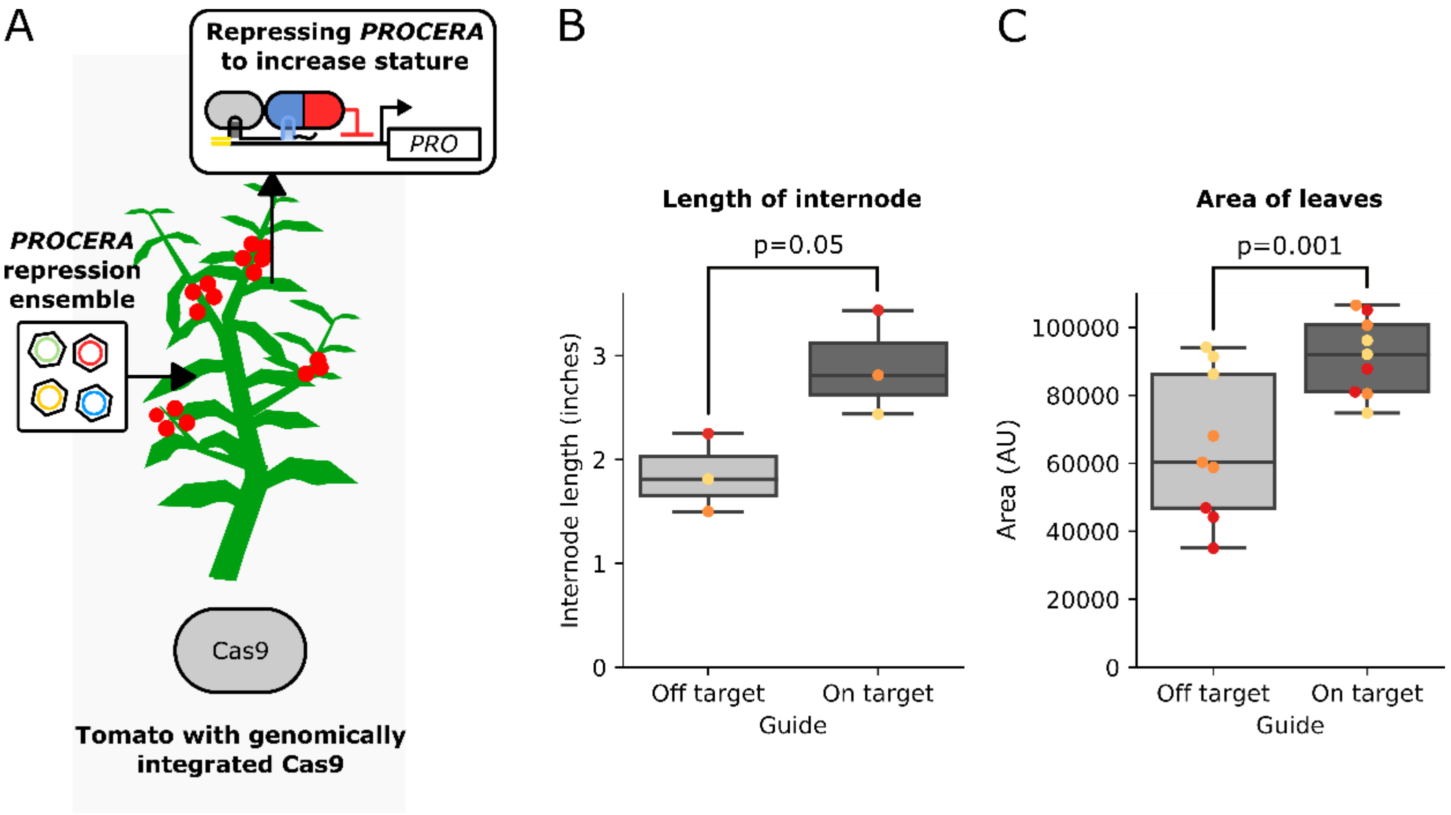

Supplemental Figure S12. Phenotypic effects of ViN 2.0 ensembles repressing *PROCERA* can be replicated in a second set of independently grown plants. A) Schematic describing the ViN 2.0 repressor ensemble used to deliver a TPLN188 repressor and sgRNA scaffolds to target it to the *PROCERA* gene in a tomato line that stably expresses Cas9. B) Boxplots summarizing the lengths of the fourth internode of the plants described in panel A treated with ViN vectors encoding off-target (light grey) or on-target (dark grey) sgRNAs. In the boxplots the horizontal line through the box marks the median, the box is the range from the 25th to the 75th percentile, and the whiskers show the highest and lowest data points within 1.5 times the interquartile range. Each dot of a different color represents data from an independent biological replicate (n=3 per treatment). Reported p-values were calculated using a t-test, and $p<0.05$ was considered significant. C) Boxplots summarizing the area of leaves of the plants described in panel A treated with ViN vectors encoding off-target (light grey) or on-target (dark grey) sgRNAs. AU, arbitrary units. In the boxplots the horizontal line through the box marks the median, the box is the range from the 25th to the 75th percentile, and the whiskers show the highest and lowest data points within 1.5 times the interquartile range. Each dot of the same color represents data from an independent biological replicate (n=3 per treatment). Reported p-values were calculated using a t-test, and $p<0.05$ was considered significant.

Supplemental Table S1. List of plasmids.

Plasmid #	Plasmid description
P38	HACKER locus 3
p145	PHD6 [p2301Y-tOCS-pUBQ1:NLS-Venus-LucPlus-tUBQ1-pU6:pUBQ1_sgRNA_Target1-tU6-pUBQ10:dCas9-RGA1-TPLRD2-tNos]
P178	HACKER locus 20
P254	TRV2-NLS-PCP-SRDX-STOP
P262	TRV2-NLS-PCP-SRDX-STOP-Ft_peptide
P263	TRV2-NLS-COM-VP64-STOP-Ft_peptide
P266	TRV2-NbDFR_Target2_truncated-sgRNAhandle-COM-Ft_peptide
P267	TRV2-NbDFR_Target3_truncated-sgRNAhandle-COM-Ft_peptide
P269	TRV2-NbPDS3.1_truncated_target_2_sgRNA-PP7
P270	TRV2-NbPDS3.1_truncated_target_2_sgRNA-PP7-Ft_peptide
P272	TRV2-NbPDS3.2_truncated_target_1_sgRNA-PP7-Ft_peptide
P295	TRV2-NLS-PCP-TPL_N188-STOP-Ft_peptide
P309	TRV2-NbPDS3.1_truncated_target_1_sgRNA-PP7
P310	TRV2-NbPDS3.1_truncated_target_1_sgRNA-PP7-Ft_peptide
P312	TRV2-NbPDS3.2_truncated_target_2_sgRNA-PP7-Ft_peptide
P383	TRV2_pAtGID1a_target_3_truncated_sgRNA-PP7-FT_peptide
P384	TRV2_pAtGID1b_target_3_truncated_sgRNA-PP7-FT_peptide
P385	TRV2_pAtGID1c_target_3_truncated_sgRNA-PP7-FT_peptide
P386	TRV2-AtPAP1_truncated_target_1_sgRNA-COM-Ft_peptide
P387	TRV2-AtPAP1_truncated_target_2_sgRNA-COM-Ft_peptide
P388	TRV2-AtPAP1_truncated_target_3_sgRNA-COM-Ft_peptide
P389	TRV2-pNbGA20ox1-1_truncated_target_1_sgRNA-PP7-Ft_peptide
P390	TRV2-pNbGA20ox1-1_truncated_target_2_sgRNA-PP7-Ft_peptide
P391	TRV2-pNbGA20ox1-2_truncated_target_1_sgRNA-PP7-Ft_peptide
P392	TRV2-pNbGA20ox1-2_truncated_target_2_sgRNA-PP7-Ft_peptide
P393	TRV2-pNbGA20ox1-3_truncated_target_1_sgRNA-PP7-Ft_peptide
P394	TRV2-pNbGA20ox1-3_truncated_target_2_sgRNA-PP7-Ft_peptide
P395	TRV2-pNbGA20ox1d-1_truncated_target_1_sgRNA-PP7-Ft_peptide
P396	TRV2-pNbGA20ox1d-1_truncated_target_2_sgRNA-PP7-Ft_peptide
P397	TRV2-pNbGA20ox1d-2_truncated_target_1_sgRNA-PP7-Ft_peptide
P398	TRV2-pNbGA20ox1d-2_truncated_target_2_sgRNA-PP7-Ft_peptide
P472	TRV2_SlProcera_Target_3_truncated_sgRNA-2xPP7-FT_peptide
P473	TRV2_SlProcera_Target_4_truncated_sgRNA-2xPP7-FT_peptide
P474	TRV2_SlProcera_Target_5_truncated_sgRNA-2xPP7-FT_peptide

Supplemental Table S2. List of sequences targeted by sgRNAs.

Guide name	Sequence
NbDFR_Target2	TATAGATAAGAAAG
NbDFR_Target3	TATGCCTTACCTTT
NbPDS1_Target1	AAATTCAAAATAGC
NbPDS1_Target2	TCAGAATATTATAC
NbPDS2_Target1	CAGGTTGGATTACG
NbPDS2_Target2	TACTTTTATTAAAA
AtGID1a_Target3	AGGGATGAGTAGGG
AtGID1b_Target3	GACCAATCGGACGG
AtGID1c_Target3	AAGAATATCGGCGT
AtPAP1_Target1	ACATTTGTCCAAAT
AtPAP1_Target2	ACCTTTGAAAATGA
AtPAP1_Target3	TAGAGCATTTTCAT
NbGA20ox1-1_Target1	GGTGTACTCAACTC
NbGA20ox1-1_Target2	TAACCCATTGGTTT
NbGA20ox1-2_Target1	AGACGAATAACTTA
NbGA20ox1-2_Target2	GCATTTCCTTTGTA
NbGA20ox1-3_Target1	TCCCGATAGTGTTT
NbGA20ox1-3_Target2	GAGACATCACCACA
NbGA20ox1D-1_Target1	GCTCGTATTATTTG
NbGA20ox1D-1_Target2	GACAGTTGGTATTG
NbGA20ox1D-2_Target1	ATATATAGACAGGA
NbGA20ox1D-2_Target2	GCATTAGTTTAGGA
SlProcera_Target3	TGGGGGAGTTTGAA
SlProcera_Target4	AGCTTCAAGAATGG
SlProcera_Target5	TCACAATCCCCCAA
*PDS1*_full_length_guide1	CAGCTTATCTTTGGAGCTCG
*PDS1*_full_length_guide2	TTGGTAGTAGCGACTCCATG

Supplemental Table S3. List of RT-qPCR primers.

NicotianaBenthamiana_Ef1alpha_qPCR-f	CTTCGTAATCCTGTTCCCTTCTC
Nicotiana_Benthamiana_Ef1alpha_qPCR-r	CACACGAAGGCCGTACTATAAG
Procera_qPCR-f	CCAACTGGGATTCTTCCTCAA
Procera_qPCR-r	CAAGCCACCACGTTACAAATC
qCAC-F	CCTCCGTTGTGATGTAACTGG
qCAC-R	ATTGGTGGAAAGTAACATCATCG
NbDFR_qPCR-f	CTGGAGCGACTTGGACTTTAT
NbDFR_qPCR-r	GCCTTCTCTGCCAGTATCTTAG
NbPDS1_qPCR-f	CAAGACCGGAGCTAGACAATAC
NbPDS1_qPCR-r	CCTGCACCAGCAATAACAATC
NbPDS2_qPCR-f	CCAAGACCAGAGCTAGACAATAC
NbPDS2_qPCR-r	ATCACCTGCACCAGCAATAA
PCP_qPCR-f	GCGTATCGCGTCAACCTAAA
PCP_qPCR-r	GTGCTATTCGCAACGATTGTC
NbGA20ox1-3_qpcr-f	TGGTGAGCACTGTGGTTATG
NbGA20ox1-3_qpcr-r	GATCCTTCCTCAGCAGAATATTGA
NbGA20ox1-1_qpcr-f	CTTGGAGAGCATTGTGGTTATG
NbGA20ox1-1_qpcr-r	GAGTCTTCTTCAGCGGAGTATC
NbGA20ox1-2_qpcr-f	GGTTGAGCCTTGGAGTTAGTAG
NbGA20ox1-2_qpcr-r	CTGTTCCTAAAGTGAGCTCTGG
NbGA20ox1D-1_qpcr-f	GAAAGCATAGGAGTGGGAAGAG
NbGA20ox1D-1_qpcr-r	TCCTGTCCCTAGAGTCAAATCA
NbGA20ox1D-2_qpcr-f	GGAGAAGGAAAGAGCCAAGAG
NbGA20ox1D-2_qpcr-r	GAAGAAGGAACCTCAGCAGAATA
AtGID1a_qPCR-f	GGAGGTAACATCGCGCATAA
AtGID1a_qPCR-r	TCTCTCATTCCCACCAAACATAG
AtGID1b_qPCR-f	ATGTTTGGTGGACAGGAGAG
AtGID1b_qPCR-r	CGGTAGATAAGCCCTCCAATAC
AtGID1c_qPCR-f	GTTTGGAGGGACCGAAAGAA
AtGID1c_qPCR-r	AGGAAGAAACGCTCTCCAATAC
AtPAP1_qPCR-f	CCTGGTCCTAATTCACCTCAAC
AtPAP1_qPCR-r	CAGTCTTAGAACCGGGCTTAAC

Supplemental Table S4. Gene accessions referenced in the manuscript.

Gene Name referenced	Accession or Identifier
NbGA20ox1-1	Niben101Scf02083g03005
NbGA20ox1-2	Niben101Scf10027g00002
NbGA20ox1-3	Niben101Scf03245g00004
NbGA20ox1D-1	Niben101Scf06024g01005
NbGA20ox1D-2	Niben101Scf06404g00009
AtGID1a	AT3G05120
AtGID1b	AT3G63010
AtGID1c	AT5G27320
AtPAP1	AT1G56650
NbPDS1	Niben101Scf14708g00023.1
NbPDS2	Niben101Scf01283g02002.1
SlGAI (PROCERA)	NM_001247436.2
NbDFR	Niben101Scf00305g05035.1
